# The National Health and Resilience in Veterans Study: A Narrative Review and Future Directions

**DOI:** 10.3389/fpsyt.2020.538218

**Published:** 2020-12-09

**Authors:** Brienna M. Fogle, Jack Tsai, Natalie Mota, Ilan Harpaz-Rotem, John H. Krystal, Steven M. Southwick, Robert H. Pietrzak

**Affiliations:** ^1^U.S. Department of Veterans Affairs National Center for Posttraumatic Stress Disorder, VA Connecticut Healthcare System, West Haven, CT, United States; ^2^Department of Psychiatry, Yale School of Medicine, New Haven, CT, United States; ^3^Department of Social and Behavioral Sciences, Yale School of Public Health, New Haven, CT, United States

**Keywords:** veterans, mental health, aging, genetics, resilience (psychological), EPI—epidemiology

## Abstract

United States (U.S.) veterans are substantially older than their non-veteran counterparts. However, nationally representative, population-based data on the unique health needs of this population are lacking. Such data are critical to informing the design of large-scale outreach initiatives, and to ensure the effectiveness of service care delivery both within and outside of the Veterans Affairs healthcare system. The National Health and Resilience in Veterans Study (NHRVS) is a contemporary, nationally representative, prospective study of two independent cohorts (*n* = 3,157 and *n* = 1,484) of U.S. veterans, which is examining longitudinal changes, and key risk and protective factors for several health outcomes. In this narrative review, we summarize the main findings of all NHRVS studies (*n* = 82) published as of June 2020, and discuss the clinical implications, limitations, and future directions of this study. Review of these articles was organized into six major topic areas: post-traumatic stress disorder, suicidality, aging, resilience and post-traumatic growth, special topics relevant to veterans, and genetics and epigenetics. Collectively, results of these studies suggest that while a significant minority of veterans screen positive for mental disorders, the majority are psychologically resilient. They further suggest that prevention and treatment efforts designed to promote protective psychosocial characteristics (i.e., resilience, gratitude, purpose in life), and social connectedness (i.e., secure attachment, community integration, social engagement) help mitigate risk for mental disorders, and promote psychological resilience and post-traumatic growth in this population.

## Introduction

Nationally representative epidemiological studies of United States (U.S.) military veterans conducted outside of the Veterans Affairs (VA) healthcare system are sparse, yet highly valuable, given that only about half (48%) of veterans utilize any VA healthcare services (1), and even fewer (~20%) utilize VA care as their primary source of health care (2). Moreover, utilization of VA healthcare services is driven by many factors, such as sex, race, income, marital status, and physical and mental health conditions (1, 2). Thus, it is critical to understand the unique needs of veterans at a broader population level to better inform the design of large-scale outreach initiatives and to help ensure the effectiveness of service care delivery.

Further, while a substantial body of research on veterans has focused on risk factors for prevalent mental and physical health conditions, few studies have assessed potentially modifiable factors that may help mitigate risk for these conditions, such as gratitude, social support, optimism, community integration, and purpose in life (3, 4). Accordingly, the overarching goal of the National Health and Resilience in Veterans Study (NHRVS) is to characterize the longitudinal trajectories of mental and physical health outcomes in U.S. veterans, and to study genetic and environmental risk and protective factors that contribute to these trajectories. The NHRVS is a large, nationally representative prospective study which consists of two separate cohorts of 3,157 and 1,484 U.S. military veterans. Additionally, the NHRVS utilizes well-validated measures to examine longitudinal changes of mental and physical health outcomes in this population.

Since the NHRVS began in 2011, it has yielded a total of 82 publications. In this review, we summarize the main findings of all NHRVS studies published as of June 2020, and discuss the broader clinical implications, limitations and future directions of this study. To thematically organize the content, the 82 articles were sorted into six major topic areas: post-traumatic stress disorder, suicidality, aging, resilience and post-traumatic growth, special topics relevant to veterans, and genetics.

## Methods

### Methodology for National Health and Resilience in Veterans Study

The NHRVS cohorts were recruited to complete a 60-min online survey from a research panel of over 50,000 households that was developed and maintained by GfK Knowledge Networks, Inc. (now Ipsos). GfK Knowledge Networks, Inc. recruited panel members through national random samples by telephone and postal mail. Internet and computer hardware access are provided if needed. GfK Knowledge Networks, Inc. uses dual sampling frames to recruit participants and offers coverage of ~98% of U.S. households. These sampling procedures are different from Internet convenience panels (i.e., “opt in” panels) as it included listed and unlisted telephone numbers, telephone and non-telephone households, cell-phone-only households, and households with and without Internet access. Only individuals sampled through these probability-based techniques are eligible to participate in the survey. To promote generalizability of results to the entire population of U.S. veterans, post-stratification weights based on demographic distributions of US veterans from concurrent US Census data (5) were applied. Of the 4,750 veterans who were in the survey panel at the time the first cohort (Cohort one; C1) was fielded, 3,408 (71.7%) completed a screening question to confirm their study eligibility (current or past active military status). Of these veterans who completed the screening question, 3,188 (93.5%) confirmed their current or past active military status, and 3,157 (92.6%) completed the survey.

C1 completed a baseline survey in 2011, which included comprehensive measures of demographics and military history, medical and psychiatric status, and psychosocial functioning. Following the baseline survey, 2-, 4-, and 7-year follow-ups were conducted. Wave 2 (*n* = 2,157; 68.3%) data were collected in 2013, Wave 3 (*n* = 1,538; 48.7%) in 2015, and Wave 4 (*n* = 1,310; 41.5%) in 2018. Additional saliva samples were collected from 500 veterans in Wave 3 for epigenetic and telomere length assays. At baseline, C1 was 90.6% male, 76.2% White, 9.6% Black, 8.2% Hispanic, 1.5% mixed races, and the mean age was 60.3 (*SD* = 15.0, range = 21–96), with 57.3% aged 60 years or older. Two-thirds of participants (66.7%) had completed at least some college education, 75.6% were married/cohabitating, 44.0% reported a household income of $60,000 or greater, 43.5% were retired, and 40.7% reported working part-time and/or full-time. The majority of did not serve in a combat or war zone (65.2%), and 19.3% reported using VA healthcare as their main source of health care.

Cohort 2 (C2) completed a baseline survey in 2013 and a follow-up survey 3 years later. A total of 1,602 individuals responded “Yes” to an initial screening question that confirmed veteran status and 1,484 participated, resulting in a response rate of 92.6%. C2 consists of 1,484 veterans who completed a baseline survey and 713 (48.0%) who completed a 3-year follow-up survey. Veterans in C2 were demographically similar to C1, with 89.7% male, 75.4% White, 9.7% Black, 9.1% Hispanic, and 1.4% mixed races, with a mean age of 60.4 (*SD* = 15.3, range 20–94) at baseline. The majority of C2 veterans were married/cohabitating (70.1%), 44.2% were retired, and 41.6% were working part-time and/or full-time, and had household incomes of $60,000 or greater (43.4%); two-thirds (66.9%) had completed at least some college education. The majority of C2 veterans did not serve in a combat or war zone (61.6%), and 21.3% reported using VA as their main source of healthcare. Saliva samples were also obtained from both NHRVS cohorts, with genetic data (PsychChip) available for 2,827 veterans.

### Review Methodology

A search of Pubmed and Scopus was conducted with the terms, “National Health and Resilience in Veterans Study”; “NHRVS”; “nationally representative sample of U.S. veterans”; “U.S. veteran population”; and the author/principal investigator “Pietrzak, R.H.” Non-NHRVS studies were excluded if “residential treatment”; “National Epidemiologic Survey on Alcohol and Related Conditions”; “Million Veteran Program”; or “VA cooperative study 504” were in the title/abstract. This search revealed 90 studies, 8 of which were excluded for not utilizing NHRVS data upon further review. The final review included 82 original research studies.

## Post-traumatic Stress Disorder

Eighteen studies (see Table 1) utilized data from the NHRVS to examine the prevalence and risk factors for PTSD and co-occurring conditions in veterans, as well as the factor and network structure of PTSD symptomatology. Below, we summarize results of these studies.

**Table 1:** Post-traumatic stress disorder.

**Citation**	**Title**	**Sample**	**Findings**	**Clinical implications**
Wisco et al. (6)	Post-traumatic stress disorder in the US veteran population: Results from the National Health and Resilience in Veterans Study	Cohort 1 (*n* = 3,157)	The prevalence of lifetime and current PTSD was 8.0 and 4.8%, respectively, and PTSD was associated with elevated risk for mood, anxiety, suicidality, and substance use disorders.	Protective characteristics such as resilience, community integration, and secure attachment style, were associated with decreased risk for PTSD, thereby suggesting the importance of potentially targeting these factors in prevention and treatment efforts.
Armour et al. (7)	Dimensional structure of DSM-5 post-traumatic stress symptoms: Support for a hybrid anhedonia and externalizing behaviors model	Cohort 2 (*n* = 1,484)	A new 7-factor model of DSM-5 PTSD symptoms was found to be a superior fit compared to 5 other models ranging between 4- and 6-factors of PTSD.	Identifying the optimal dimensional representation of PTSD symptoms can help inform the etiology of PTSD, and comorbidity between PTSD and other psychiatric disorders.
Pietrzak et al. (8)	Functional significance of a novel 7-factor model of DSM-5 PTSD symptoms: Results from the National Health and Resilience in Veterans study	Cohort 2 (*n* = 1,484)	Differential patterns of associations were observed between 7 DSM-5 PTSD symptom clusters, and psychiatric comorbidities, suicidal ideation, hostility, and functioning and quality of life.	Results suggest that a 7-factor hybrid model of DSM-5 PTSD symptoms provides greater specificity in regards to how the phenotypic expression of PTSD symptoms relates to comorbidities, suicidality, hostility, and functioning and quality of life.
Tsai et al. (9)	Dissociative subtype of DSM-5 post-traumatic stress disorder in U.S. veterans	Cohort 2 (*n* = 1,484)	19.2% of veterans with lifetime PTSD and 16.1% of veterans with past-month PTSD screened positive for the dissociative subtype. Those with the dissociative subtype reported more severe PTSD symptoms, comorbid depressive and anxiety symptoms, alcohol use problems.	It is clinically relevant to identify veterans with the dissociative subtype of PTSD, as they reported higher levels of depression, anxiety, alcohol problems, hostility, and PTSD symptoms.
Tsai et al. (10)	Dimensional structure of DSM-5 post-traumatic stress disorder symptoms: Results from the National Health and Resilience in Veterans Study	Cohort 2 (*n* = 1,484)	5.2% of veterans screened positive for past-month PTSD and a new 6-factor model of DSM-5 PTSD symptoms was found to provide the best dimensional representation of PTSD symptom clusters and demonstrated validity in assessing health outcomes.	Findings suggest the 6-factor model of PTSD symptoms may provide a more refined phenotypic model of PTSD than the 4-factor DSM-5 model, and may have utility in understanding the etiology of PTSD, personalization of treatment, and monitoring of treatment outcomes.
Contractor et al. (11)	Latent profiles of DSM-5 PTSD symptoms and the “Big Five” personality traits	Cohort 2 (*n* = 1,226)	Comparing all classes to the most severe PTSD class, most differences were found for utilization of mental health treatment; and the preferred use of self-distraction, denial, active coping, emotional and instrumental support, and behavioral disengagement coping strategies.	Findings underscore the importance of considering personality pathology in examining the heterogeneity of PTSD symptom presentation, as typologies differed in relation to mental healthcare utilization, coping strategies, and substance use.
Mota et al. (12)	Late-life exacerbation of PTSD symptoms in US veterans: Results from the National Health and Resilience in Veterans Study	Cohort 1 Baseline (*n* = 2,119) and Wave 2 (*n* = 1,441)	Approximately 1 in 10 older US veterans experienced a clinically significant exacerbation of PTSD symptoms in late life. Executive dysfunction may have contributed to risk for exacerbated PTSD symptoms.	Results underscore the importance of routine assessment and monitoring of PTSD symptoms, particularly anxious arousal, in aging veterans, even among those who have not previously been symptomatic.
Mota et al. (13)	High burden of subthreshold DSM-5 post-traumatic stress disorder in U.S. military veterans	Cohort 2 (*n* = 1,484)	Approximately one in three US veterans experienced clinically significant PTSD symptoms in their lifetime. Further, subthreshold PTSD was associated with elevated comorbid psychiatric disorders, as well as decrements in mental and physical functioning.	Results underscore the importance of assessment, prevention and treatment efforts in targeting veterans and other trauma-affected individuals clinically elevated PTSD symptoms that are below the threshold for a diagnosis of PTSD.
Wisco et al. (14)	Probable post-traumatic stress disorder in the US veteran population according to DSM-5: Results from the National Health and Resilience in Veterans Study	Cohort 2 (*n* = 1,484)	The prevalence of DSM-5 probable PTSD, conditional probability of probable PTSD, and odds of psychiatric comorbidity were similar to prior findings with DSM-IV-based measures in US veterans.	Findings indicate that veterans should be assessed for a wide range of both military and non-military trauma when screened for PTSD, and highlight the high rates of comorbid PTSD and other mental disorders.
Armour et al. (15)	A network analysis of DSM-5 post-traumatic stress disorder symptoms and correlates in U.S. military veterans	Cohort 2 (*n* = 221)	The 20-item DSM-5 PTSD network demonstrated that the most central symptoms were negative trauma-related emotions, flashbacks, detachment, and physiological cue reactivity.	Specific intrusive, negative cognition and mood, and hyperarousal symptoms are central to the PTSD symptom network in symptomatic veterans. Interventions targeting these symptoms may help mitigate chronicity of this disorder.
Claycomb Erwin et al. (8)	The 7-factor hybrid model of DSM-5 PTSD symptoms and alcohol consumption and consequences in a national sample of trauma-exposed veterans	Cohort 2 (*n* = 916)	CFA results indicated an excellent fit for the combined CFA model of the 7-factor hybrid model of PTSD and two-factor AUDIT model. Lifetime dysphoric arousal, negative affect, and anhedonia symptom clusters were most strongly associated with past-year alcohol consequences.	Results highlight the need to assess for alcohol-related consequences in veterans with internalizing PTSD symptoms, and underscore the importance of assessing beyond mere alcohol consumption to the adverse consequences of alcohol use.
Wisco et al. (16)	A comparison of ICD-11 and DSM criteria for post-traumatic stress disorder in two national samples of U.S. military veterans	Cohort 1 (*n* = 3,157) and Cohort 2 (*n* = 1,484)	A significantly greater proportion of veterans met criteria for lifetime and past-month PTSD under DSM-IV/5 than under ICD-11. 21.8–35.9% of those who met criteria under DSM IV/5 did not meet under ICD-11, whereas only 2.4–7.1% of those who met under ICD-11 did not meet under DSM-IV/5.	Of the estimated 1.3 million U.S. veterans with a lifetime history of DSM-5 PTSD, over 280,000 (22%) would not meet ICD-11 criteria and these individuals might not have access to the same healthcare, disability benefits, and other important services that they would under DSM-5
Wolf et al. (17)	The Dissociative Subtype of PTSD Scale: Initial evaluation in a national sample of trauma-exposed veterans	Resampled subset from Cohort 1 (*n* = 860)	Of the veterans with possible lifetime PTSD, nearly 40% of were included in the subtype. Items assessing derealization and depersonalization were the strongest indicators of the dissociative subtype.	Derealization/depersonalization on the DSPS has the potential to identify individuals with the dissociative subtype of PTSD, while the amnesia and loss of awareness subscales may provide useful supplemental Information about current symptoms.
von Stockert et al. (18)	Evaluating the stability of DSM-5 PTSD symptom network structure in a national sample of U.S. military veterans	Cohort 2 Baseline and Wave 2 (*n* = 611)	PTSD symptoms at Time 1 and Time 2 using the DSM-5 PTSD symptom network structure did not differ significantly with regard to network structure or global strength.	Findings suggest that trauma-related avoidance, intrusive, and dysphoric symptoms may contribute to the chronicity of PTSD symptoms in veterans.
Byrne et al. (19)	Latent typologies of DSM-5 PTSD symptoms in U.S. military veterans	Cohort 2 (*n* = 158)	A three-class solution was determined, with Dysphoric (36.2%), High Symptom (34.0%), and Threat (29.8%). Threat had greater intrusions and avoidance; Dysphoric had greater negative affect. The High Symptom typology had high probabilities of all symptoms.	The three identified PTSD typologies were differentially linked to clinical and trauma characteristics. These findings underscore the importance of a personalized approach to the assessment and treatment of DSM-5 PTSD.
Kachadourian et al. (20)	Post-traumatic stress disorder symptoms, functioning, and suicidal ideation in US military veterans: A symptomics approach	Cohort 2 (*n* = 1,484)	Nonspecific anhedonic and hyperarousal symptoms of PTSD were most strongly associated with measures of physical and mental functioning, quality of life, and suicidal ideation.	Interventions targeting non-specific anhedonic/internalizing and irritability and aggression symptoms of PTSD may be useful in mitigating suicidal ideation, and promoting functioning and quality of life in veterans.
McCarthy et al. (21)	Self-assessed sleep quality partially mediates the relationship between PTSD symptoms and functioning and quality of life in U.S. veterans: Results from the National Health and Resilience in Veterans Study	Cohort 1 (*n* = 3,157)	27.6% of veterans reported poor sleep quality, with poor sleep quality being significantly higher among veterans who screened positive for probable PTSD. Poor sleep quality partially mediated the relationships between PTSD symptom severity and functioning and QOL measures.	Nearly 1 of 3 U.S. veterans reported poor quality sleep. Assessment and monitoring of sleep difficulties may help mitigate the deleterious effect of PTSD symptoms on functioning and QOL.
Herzog et al. (22)	Dissociative symptoms in a nationally representative sample of trauma-exposed U.S. military veterans: Prevalence, comorbidities, and suicidality	Cohort 2 (*n* = 1,264)	1 in 5 veterans endorsed mild-to-severe dissociative symptoms and veterans with dissociative symptoms had greater psychiatric comorbidity and poorer functioning. Dissociative symptoms predicted suicide risk above other comorbidities and trauma history.	Dissociative symptoms in veterans may be a transdiagnostic risk factor for mental disorders and functional difficulties independent of trauma exposure and PTSD.

### Prevalence

PTSD is one of the most prevalent mental disorders among U.S. veterans. Prevalence estimates of this disorder among veterans ranging widely (23, 24), partly due to most studies being conducted in non-representative samples (e.g., convenience sample of Vietnam-era veterans or veterans deployed to Iraq or Afghanistan). Using data from the NHRVS, which surveyed a nationally representative sample of U.S. veterans, Wisco et al. (6, 14) found that estimates of lifetime DSM-IV and DSM-5 PTSD were 8.0 and 8.1%, respectively, and past-month PTSD were 4.8 and 4.7%, respectively. Further, to understand the burden of subthreshold manifestations of PTSD, Mota et al. (13) analyzed NHRVS data and found that the prevalence of lifetime and current subthreshold DSM-5 PTSD was 22.1 and 13.5%, respectively. These prevalences were markedly higher than prevalences of lifetime and current PTSD (8.0 and 4.5%, respectively) (13).

### Exacerbation/Re-emergence of PTSD in Late-Life

Research on older veterans has also suggested that PTSD symptoms may re-emerge or become exacerbated in older age among trauma-exposed veterans (25). Using NHRVS data, Mota et al. (12) found that nearly 10% of older U.S. veterans experienced exacerbated PTSD symptoms an average of nearly 3 decades after their worst trauma. Cognitive difficulties, particularly perceived deficits in executive function, were the sole determinant of late-life exacerbation of PTSD symptoms. These findings suggest that late-life exacerbation of PTSD symptoms affects a substantial minority of older veterans in the U.S., and that reductions in executive control may increase risk for this phenomenon.

### Longitudinal Trajectories of PTSD Symptoms

A recent study (26) utilized all four waves of data from C1 and found evidence of three predominant courses of PTSD symptoms over a 7-year period: low/no (89.2%), moderate (7.6%), and severe (3.2%). Relative to veterans in the no/low symptom course, those in the moderate and severe courses endured a significantly greater number of lifetime traumatic events, reported greater physical health difficulties, were more likely to have lifetime psychiatric histories; and reported lower social connectedness. Importantly, more than 10% veterans evidenced a symptomatic elevation of PTSD symptoms that steadily declined over a 7-year period. Results suggested that interventions to help bolster social connectedness may help mitigate risk for symptomatic trajectories of PTSD in U.S. veterans.

### DSM-5 vs. ICD-11 Classification of PTSD

The *Diagnostic and Statistical Manual of Mental Disorders* (DSM) and *International Classification of Diseases* (ICD) are the two major diagnostic systems commonly used worldwide and, historically, the two have defined PTSD using similar criteria. However, in the recent publication of the ICD-11 (27), a definition of PTSD was proposed that diverges substantially from DSM-5 and reduces the total number of symptoms to 6, compared to 20 in DSM-5. Therefore, it is important to compare how using different diagnostic definitions of PTSD may impact reported prevalence of the disorder. To evaluate this possibility, Wisco et al. (16) found that using the ICD-11 diagnostic criteria yielded significantly lower estimates of lifetime (6.9 vs. 5.0%) and past-month (4.0 vs. 2.7%) PTSD than DSM-5 criteria, without reducing associations with psychiatric comorbidities. Importantly, among individuals excluded under ICD-11, all endorsed clinically significant distress or functional impairment related to their PTSD symptoms, suggesting that the ICD-11 criteria may underestimate clinically meaningful distress and impairment related to PTSD symptoms (16).

### Dissociative Subtype of PTSD

The DSM-5 formally introduced a dissociative subtype of PTSD, which includes symptoms of depersonalization (i.e., “the experience of being an outside observer of or detached from oneself”) and/or derealization [i.e., “the experience of unreality, distance, or distortion; (28)]. Tsai et al. (9) evaluated the prevalence and correlates of this subtype of PTSD in the NHRVS. They found that 19.2 and 16.1% of veterans with a positive screen for lifetime and past-month DSM-5 PTSD, respectively, also screened positive for the dissociative subtype. In 2017, Wolf et al. (17) developed and evaluated the Dissociative Subtype of PTSD Scale (DSPS) using a resampled subset of C1 from the NHRVS, and found that nearly 40% of those who screened positive for lifetime PTSD met criteria for the dissociative subtype. Importantly, dissociative symptoms may not exclusively occur in the context of a PTSD diagnosis. A recent NHRVS study (22) found that 20.8% of trauma-exposed veterans experience dissociative symptoms, even if they do not screen positive for this disorder. Results of this study suggest that dissociative symptoms, independent of a PTSD diagnosis, may be a transdiagnostic risk factor for mental health disorders and poor functioning in veterans (22).

### PTSD Symptoms and Functioning

PTSD deleteriously affects various aspects of functioning, including physical health, quality of life (QOL), and psychosocial functioning. Given that there is considerable variability in PTSD symptom presentation among trauma survivors, analysis of individual symptoms that are associated with measures functioning may provide more nuanced insight into how this heterogeneous syndrome may impact these outcomes. Using NHRVS data, Kachadourian et al. (20) employed a novel “symptomics” approach to identify specific PTSD symptoms linked to functional difficulties in trauma-exposed veterans. They found that the non-specific symptoms of this disorder (e.g., difficulty experiencing positive affect, sleep difficulties, loss of interest) were the strongest correlates of poor functioning and suicidal thinking. Furthermore, McCarthy et al. (21) found that sleep difficulties may partially mediate the relationship between PTSD symptoms, and functioning and QOL.

### Symptom Structure of PTSD

The DSM-5 criteria for PTSD include four symptom clusters of intrusions, avoidance, negative alterations in cognitions and mood, and alterations in arousal and reactivity (28). Tsai et al. (10) found that a 5-factor dysphoric arousal model and a 6-factor externalizing behavior model provided a better fit than the 4-factor model of DSM-5 PTSD symptom clusters in C2 of the NHRVS, with the externalizing behavior model providing the best dimensional representation of the symptom clusters. Expanding on this work, a novel 7-factor hybrid model (7) of PTSD was proposed utilizing NHRVS data. This model incorporated unique features of the 6-factor externalizing behavior model (10) with those of another 6-factor (29) to suggest a 7-factor model of intrusions, avoidance, negative affect, anhedonia, externalizing behaviors, anxious arousal (i.e., hypervigilance, exaggerated startle response), and dysphoric arousal (i.e., sleep and concentration difficulties) symptoms. This more nuanced structural model was found to be the best-fitting structural model of DSM-5 PTSD symptoms in veterans and a sample of university students (7). Since the publication of this 7-factor model of DSM-5 PTSD symptoms, several additional studies in various trauma-exposed samples have found support for this structural model of PTSD [e.g., (30–34)].

Evaluating the underlying structure of PTSD symptoms allows researchers to determine specific sets of symptoms that account for comorbidity of PTSD with other disorders and aspects of functioning. Using NHRVS data, Pietrzak et al. (8) examined the functional significance of the 7-factor hybrid model with comorbid psychopathology (e.g., depression, anxiety, suicide ideation, hostility) and found differential patterns of associations between PTSD symptom clusters and comorbidities. Specifically, anhedonia symptoms were most strongly related to current depression, reduced mental functioning, and quality of life. Externalizing behaviors were most strongly related to hostility. Further, dysphoric arousal, negative affect, and anhedonia symptom clusters were most strongly associated with past-year alcohol consequences (35).

Data from the NHRVS have also been used to examine latent profiles of DSM-5 PTSD symptoms. One study (19) found a three-class solution, described as Dysphoric (i.e., high probabilities of negative affect and anhedonia symptoms), Threat (i.e., high probabilities of intrusive and avoidance symptoms), and High Symptom (i.e., high probabilities of all PTSD symptoms). A related study (11) found evidence of a 5-class PTSD-personality typology solution, which differed with respect to DSM-5 PTSD symptom cluster severity and several of the “Big Five” personality dimensions. Both studies found that these typologies were differentially related to clinical and trauma characteristics, thus underscoring the importance of considering differential “person-based” manifestations of PTSD symptomatology in personalized approaches to the assessment and treatment of this disorder.

### Network Structure of PTSD Symptoms

Exposure to traumatic events in veterans often involves life-threatening combat, injuries, accidents, loss, or interpersonal violence, such as sexual trauma (6). While initial symptoms, such as trouble sleeping, are considered to be a normal reaction to stress in the short term and many veterans manage to overcome these symptoms over time (36), more persistent symptoms can be debilitating. PTSD is a characterized by heterogeneous constellation of symptoms, including intrusive memories related to the trauma, hypervigilance to and avoidance of trauma-related situations and memories, and negative cognitions and mood (28). Network theory suggests that symptoms are correlated in a syndrome because they directly activate and dynamically interact with each other, rather than because they have a shared origin (15). However, until recently little was known about the network structure of PTSD symptoms.

To address this gap, a network analysis of DSM-5 PTSD symptoms was conducted in participants from C2 of the NHRVS with subthreshold or greater PTSD symptoms. Results revealed that negative emotions related to the trauma, detachment, flashbacks, and physiological reactivity were most central and interconnected within the PTSD symptom network (37). Strong connections were observed between flashbacks and nightmares related to the trauma, hypervigilance and exaggerated startle response, and detachment and restricted affect (37). A follow-up study (18) evaluated the temporal stability of this network structure over a 3-year period to help identify symptoms that contribute to the chronicity of PTSD. Results indicated that the network structure for DSM-5 PTSD was stable over the 3-year period with respect to both network structure and global strength (18). Similar to prior research, avoidance, intrusive, and negative cognition and mood symptoms were among the more central nodes, suggesting these symptoms may contribute to the chronicity of PTSD in symptomatic veterans.

### Clinical Implications

Results of NHRVS studies focused on PTSD suggest that veterans report exposure to a wide range of potentially traumatic events from military and non-military experiences, and a strikingly high proportion of veterans—approximately one in three—experience clinically significant PTSD symptoms in their lifetime, with a significant minority screening positive for PTSD (6, 13, 14). Collectively, these results suggest that sleep, avoidance, intrusive, and dysphoric symptoms may contribute to the chronicity of PTSD symptoms in veterans, and may thus represent important targets in prevention, treatment, and risk reduction efforts (18, 20, 21, 37). Utilizing a 7-factor hybrid model of PTSD symptoms may also provide greater specificity in understanding how PTSD symptoms relate to mental health, functioning, and QOL in U.S. veterans (7, 8). Furthermore, resilience, social support, secure attachment, and community integration are potentially modifiable factors that are linked to decreases in PTSD symptoms and may be targeted in prevention and treatment efforts (6, 26).

## Suicidality

Suicide has been a significant public health problem among veterans for over a decade and is a top clinical priority for the VA. In 2016, the rate of deaths by suicide was 1.5 times greater for veterans than for non-veteran adults, after adjusting for age and gender (38). Numerous studies have examined potential mental and physical health risk factors for suicide in veterans, but less research has sought to identify modifiable protective factors (39). To date, seven studies (see Table 2) from the NHRVS have examined the prevalence and correlates of suicidality and identified potential protective factors in the U.S. veteran population.

**Table 2:** Suicidality.

**Citation**	**Title**	**Sample**	**Findings**	**Clinical implications**
Smith et al. (40)	Nature and determinants of suicidal ideation among U.S. veterans: Results from the National Health and Resilience in Veterans Study	Cohort 1 Baseline and Wave 2 (*n* = 2,157)	13.7% of U.S. veterans had chronic, onset, or remitted suicidal ideation. Medical and psychological co-morbidities increase risk of chronic suicidal ideation. Social connectedness was protective for remitted and onset, but not chronic suicidal ideation.	Bolstering social connectedness may be helpful in reducing risk for SI and possibly promoting remission from SI.
Pietrzak et al. (41)	Factors protecting against the development of suicidal ideation in military veterans	Cohort 1 Baseline, Wave 2, and Wave 3 (*n* = 2,093)	Decreased risk of suicidal ideation was independently associated with greater social support, curiosity, resilience, and acceptance-based coping. Increased risk of suicidal ideation was associated with loneliness, disability in instrumental daily activities, PTSD symptoms, somatic problems, alcohol use problems, denial-based coping, and higher age.	Greater perceived social support, curiosity, resilience, and acceptance-based coping are modifiable and addressed in contemporary cognitive-behavioral psychotherapies, and thus may be promising targets in prevention efforts designed to mitigate suicide risk in veterans.
Wisco et al. (42)	Moral injury in U.S. combat veterans: Results from the National Health and Resilience in Veterans Study	Cohort 2 (*n* = 564)	A total of 10.8% of combat veterans acknowledged transgressions by self, 25.5% endorsed transgressions by others, and 25.5% endorsed betrayal, which were associated with risk for mental disorders and suicidality.	Results underscore the importance of moral injury as a risk factor for mental disorders and suicidality in combat veterans. Treatments addressing moral injury may help mitigate risk for these outcomes.
Monteith et al. (43)	Psychiatric and interpersonal correlates of suicide ideation in military sexual trauma survivors: The National Health and Resilience in Veterans Study	Cohort 2 (*n* = 115)	Military sexual trauma survivors who reported more severe psychological distress, hazardous alcohol use, and perceived general disapproval from others were significantly more likely to report suicidal ideation in the past two weeks. Hazardous alcohol use and perceived general disapproval from others were associated with being more likely to report attempting suicide in adulthood.	Assessing alcohol use, beliefs regarding how one is perceived by others after their worst traumatic experience, and current psychological distress may be useful targets of screening when considering suicide risk in MST survivors.
Corona et al. (44)	Meaning in life moderates the association between morally injurious experiences and suicide ideation among U.S. combat veterans: Results from the National Health and Resilience in Veterans Study	Cohort 2 (*n* = 564)	Interactions between global meaning in life, transgressions by others, and betrayal experiences were significant. Higher meaning in life was associated with significantly lower likelihood of experiencing suicide ideation at higher levels of transgression by others and betrayal experiences.	Purpose and meaning in life may be intervention targets to help reduce suicide risk for veterans who experienced potentially morally injurious experiences of betrayal and transgressions by others.
Kachadourian et al. (45)	Protective correlates of suicidality among veterans with histories of post-traumatic stress disorder and major depressive disorder: Results from the National Health and Resilience in Veterans Study	Cohort 1 (*n* = 577)	Current suicidal ideation was 29.4% and the prevalence of lifetime SA was 28.0% in US veterans. Greater purpose in life, curiosity, and optimism were negatively associated with suicidal ideation. None of the protective correlates were associated with attempt history.	Purpose in life, curiosity, and optimism may help mitigate suicide risk and could be targeted in suicide prevention and interventions efforts in high-risk populations.
Straus et al. (46)	Purpose in life and conscientiousness protect against the development of suicidal ideation in U.S. military veterans with PTSD and MDD: Results from the National Health and Resilience in Veterans Study	Cohort 1 Baseline, Wave 2, Wave 3, and Wave 4 (*n* = 222)	Nearly one in three (27.1%) of veterans with PTSD and/or MDD developed suicidal ideation over the 7-years. Racial minority status and lower scores on measures of purpose in life, conscientiousness, and frequency of religious service attendance were independently associated with incident suicidal ideation.	Lower purpose in life and conscientiousness, which are potentially modifiable, explained the vast majority of variance in incident suicidal ideation and may be targeted in suicide prevention efforts in veterans with PTSD and MDD.

### Prevalence and Longitudinal Courses of Suicidality

Suicidal ideation (SI) is often a precursor to a suicide attempt or death by suicide. Thus, there is a great need to systematically understand the nature and prevalence of SI over time among veterans. This may provide insight into the predominant patterns and causes of SI, which can help determine targets for prevention and treatment. In 2017, Smith et al. (40) analyzed data from C1 of the NHRVS and found that 13.7% of veterans had chronic, new-onset, or remitted SI over a 2-year period. A key finding was that while mental and physical health problems were risk factors for chronic and onset SI, greater social connectedness (e.g., secure attachment style and perceived social support), was negatively related to these outcomes. Building on this work using 4-year prospective data from C1 of the NHRVS, Pietrzak et al. (41) conducted a prospective cohort study of U.S. veterans without SI at baseline to identify the incidence and baseline determinants of new-onset SI. Results revealed that greater age, higher loneliness, disability in instrumental activities of daily living, PTSD, alcohol use problems, and somatic symptoms, and use of denial-based coping were associated with increased risk for SI. After controlling for these risk factors, greater perceived social support, curiosity, resilience, and acceptance-based coping emerged as significant protective factors for SI, accounting for more than 40% of the total variance in predicting SI risk. These findings underscore the importance of considering both risk and protective factors in population-based suicide risk prevention efforts in veterans.

The risk of suicide is even greater in veterans who have been diagnosed with PTSD and/or major depressive disorder (MDD). A recently published NHRVS study (45) found that 29.4% of veterans with PTSD and/or MDD reported current SI and that 28.0% reported a lifetime suicide attempt. Greater purpose in life, curiosity, and optimism were inversely associated with SI. Subsequently, another study (46) used NHRVS data to prospectively examine how a broad range of risk and protective factors contributed to the development of SI over 7 years in this high-risk subpopulation. Importantly, 27.1% of veterans with PTSD and/or MDD who did not endorse SI at baseline developed SI during the course of 7 years of the study. Lower levels of purpose in life, conscientiousness, and frequency of religious service attendance were most strongly associated with developing SI. These findings help to characterize potential targets for population-based prevention and treatment efforts that may help mitigate suicidality in high-risk veterans.

### Combat-Related Moral Injury and Suicidality

Another military-related stressor that has been associated with elevated risk for suicide is moral injury. Moral injury is defined as “the psychological, biological, spiritual, behavioral, and social impact of perpetrating, failing to prevent, or bearing witness to acts that transgress deeply held moral beliefs and expectations” [(47), p. 698], and often includes feelings of guilt and shame (47). Moral injury can occur by transgressions by self (e.g., killing a child), transgressions by others (e.g., witnessing torture committed by others), and betrayal experiences (e.g., perceived failures by leadership or fellow service members). Using NHRVS data, Wisco et al. (42) found that 10.8–25.5% of U.S. combat veterans reported being exposed to potentially morally injurious experiences. In particular, transgressions by self were found to be associated with current mental disorders and SI, and betrayal with post-deployment suicide attempts, even after adjustment for severity of combat exposure. Building upon this work, Corona et al. (44) investigated whether global purpose and meaning in life moderated the relationship between potentially morally injurious experiences and SI in combat veterans. Results revealed that greater global purpose and meaning in life was associated with significantly lower likelihood of experiencing SI among veterans who reported higher levels of transgression by others and betrayal experiences.

### Military Sexual Trauma and Suicidality

Effectively responding to suicide risk among veterans involves further understanding reactions to military-related stressors, including military sexual trauma (MST). Veterans with a history of MST (i.e., sexual assault and/or sexual harassment during service) are at elevated risk for suicide. Accordingly, Monteith et al. (43) sought to identify psychiatric and interpersonal correlates of suicidal ideation and suicide attempt among NHRVS veterans with a history of MST. The study found MST survivors who reported more severe alcohol use problems and perceived general disapproval from others in relation to their worst traumatic event were significantly more likely to report current SI and a lifetime suicide attempt; psychological distress was additionally linked to current SI.

### Clinical Implications

Collectively, results of NHRVS studies on suicidality conducted to date indicate that a considerable proportion of U.S. veterans experience SI, and that SI courses may fluctuate over time. These findings underscore the importance of periodic monitoring of suicidal thoughts and behaviors in this population (40). Further, prevention and treatment efforts designed to mitigate psychiatric and physical health comorbidities, loneliness, and disability in instrumental activities of daily living, and bolster social connectedness (i.e., secure attachment style and perceived social support) and protective psychosocial characteristics (i.e., curiosity, resilience, acceptance-based coping) may help mitigate risk for SI in veterans (40, 41). In high-risk veterans with comorbid PTSD/MDD, higher levels of purpose in life, curiosity, conscientiousness, and optimism were associated with decreased risk of SI (45, 46). Finally, results of these studies highlight the important of routine assessment of MST and potentially morally injurious experiences in the assessment, monitoring, and treatment of suicidality in veterans.

## Aging

More than half (65%) of veterans are currently aged 55 or older (48) and it is projected that this proportion of aged veterans will increase over the next two decades (49). There is an new focus potentially modifiable protective psychosocial characteristics in veterans, such as resilience, optimism, and religiosity, which may help older persons adapt to negative life events such as medical and psychiatric illness and promote successful aging (50). To date, twelve studies (see Table 3) focusing on aging-related topics have been published utilizing NHRVS data. The majority of these studies have examined characteristics that may help promote successful aging in veterans.

**Table 3:** Aging.

**Citation**	**Title**	**Sample**	**Findings**	**Clinical implications**
Fanning and Pietrzak (51)	Suicidality among older male veterans in the United States: Results from the National Health and Resilience in Veterans Study	Cohort 1 (*n* = 1,962)	Six percent of veterans reported past 2-week suicidal ideation, and combat veterans were more likely to contemplate suicide than non-combat veterans.	The low rate of treatment among veterans contemplating suicide underscores the need to improve outreach to at-risk older veterans in the general veteran population.
Pietrzak and Cook (52)	Psychological resilience in older U.S. veterans: Results from the National Health and Resilience in Veterans Study	Cohort 1 (*n* = 2,025)	Among older U.S. veterans who endured a high number of traumas in their lifetimes, nearly 70% were psychologically resilient in later life.	Lack of disability, secure attachment style, college/higher education, emotional stability, community integration, and purpose in life were strongly related to resilience. Population-based interventions targeting modifiable factors such as community integration and purpose in life may help promote resilience in veterans.
Kuwert et al. (53)	Loneliness among older veterans in the United States: Results from the National Health and Resilience in Veterans Study	Cohort 1 (*n* = 2,025)	44% of veterans reported feeling lonely at least some of the time. The largest magnitude associations were observed for support and secure attachment style negatively associated with loneliness, and depressive symptoms being positively associated.	Results underscore the importance of multifactorial approaches (such as health and psychosocial approaches) in the prevention and treatment of loneliness in older veterans.
Levy et al. (54)	Lower prevalence of psychiatric conditions when negative age stereotypes are resisted	Cohort 1 (*n* = 2,031)	The prevalence suicidal ideation, anxiety, PTSD was found to be significantly lower among participants who fully resisted negative age stereotypes, compared to those who fully accepted them.	Findings underscore the importance of taking negative age stereotypes into account in etiological models of late-life psychiatric conditions.
Monin et al. (55)	From serving in the military to serving loved ones: Unique experiences of older veteran caregivers	Cohort 1 (*n* = 2,025)	Among the 20.4% of veteran caregivers, combat exposure was associated with less emotional caregiving strain and grandparenting was associated with increased perception of caregiving reward. Gratitude, happiness, and social support were additionally associated with greater reward.	Results suggest that significant early-life stressors may help confer greater resilience to the emotional demands of caregiving in older age and that both the caregiving context (i.e., hours, relationship type) and caregivers' attitudes may contribute to strain and reward.
Pietrzak et al. (56)	Successful aging among older veterans in the United States	Cohort 1 (*n* = 2,025)	Most older veterans rated themselves as aging successfully. Physical health difficulties and current psychological distress were most strongly negatively related to successful aging, while resilience, gratitude, and purpose in life, were most strongly positively related.	A dimensional approach to studying successful aging may help inform the development of clinical assessment, prevention, and treatment strategies that go beyond symptom management and work toward promoting functioning and well-being in older veterans.
Blais et al. (57)	Barriers and facilitators related to mental health care use among older veterans in the United States	Cohort 1 (*n* = 2,025)	Current utilization was associated with lifetime PTSD or depression, lifetime drug use disorder, severity of current psychiatric symptoms, medical difficulties, and lower perceptions of stigma.	Results highlight the importance of efforts to identify veterans who may benefit from mental health care because of predisposing and need characteristics, such as mental and physical health disorders.
Rozanova et al. (58)	Perceptions of determinants of successful aging among older U.S. veterans: results from the National Health and Resilience in Veterans Study	Cohort 1 (*n* = 2,025)	Older U.S. veterans emphasized health behaviors, social engagement, and dispositional characteristics as key determinants of successful physical, emotional, and cognitive aging	Older veterans emphasize individual health behaviors, self-reliance, and social connectedness as key determinants of successful aging. Prevention efforts to promote these factors may help promote successful aging
Mota et al. (59)	Purpose in life is associated with a reduced risk of incident physical disability in aging U.S. military veterans	Cohort 1 Baseline and Wave 2 (*n* = 1,686)	Older age, being married/cohabiting, retirement, and number of medical conditions were associated with an increased risk of any incident disability. Purpose in life was found to be protective for incident disability.	Purpose in life may help mitigate risk of physical disability through a broad range of mechanisms. Further research should evaluate whether interventions to increase purpose in life may help mitigate risk for physical disability among older veterans.
Weiner et al. (60)	Age differences in the association of social support and mental health in male U.S. veterans: Results From the National Health and Resilience in Veterans Study	Cohort 1 (*n* = 619)	Higher perceived support was associated with fewer mental health difficulties in younger but not older veterans, while community integration was associated with fewer mental health difficulties in older but not younger veterans.	Interventions targeting general mental health or global distress in younger male veterans should focus on enhancement of close relationships and feelings of support; whereas interventions focused on older male veterans should encourage integration in the community.
Monin et al. (61)	Older age associated with mental health resiliency in sexual minority US veterans	Cohort 1 (*n* = 3,095)	Younger LGB veterans were most likely to report lifetime depression and/or PTSD and current depression compared with heterosexual and older LGB veterans. Older LGB veterans had low levels of mental health problems, but reported the smallest social support networks.	Engaging older LGB veterans as a social support resource (e.g., mentoring) to help improve and/or protect the mental health of younger LGB veterans may also have positive effects for the older LGB mentors by increasing their social resources.
Levy et al. (62)	Active coping shields against negative aging self-stereotypes contributing to psychiatric conditions	Cohort 1 Baseline, Wave 2, and Wave 3 (*n* = 3,157)	Veterans with more negative age stereotypes were more likely to develop psychiatric conditions. Active coping decreased the likelihood of older individuals experiencing a detrimental health impact from negative age stereotypes.	Active coping may help blunt the adverse impact of negative age stereotypes, thus suggesting that efforts to promote adaptive coping skills may help mitigate risk for psychiatric conditions in older veterans.

### Successful Aging

Using data from C1 of the NHRVS, Pietrzak et al. (56) found that the majority (82%) of 60–96 year old veterans rated themselves as aging successfully, and that physical and mental health difficulties were most strongly negatively related to successful aging. Additionally, after adjustment for these risk factors, resilience, gratitude, purpose in life, and community integration were strongly positively related to successful aging. Subsequently, Rozanova et al. (58) qualitatively evaluated veterans' perceptions of factors important to successful aging. Results of this study resonated with those of the quantitative study, suggesting that older veterans emphasize health behaviors, social engagement, and dispositional characteristics as key determinants of successful aging.

### Purpose in Life and Physical Disability

Physical disability is an important aspect of aging that may negatively affect functioning and quality of life in older veterans. Accordingly, elucidation of modifiable factors that may help buffer against the development of physical disability are critical to further understanding determinants of successful aging. Mota et al. (59) found that over 2 years, the incidence of new-onset physical disability among veterans aged 55 years and older was 11.5%. Importantly, purpose in life, which may be modified using adjunctive intervention strategies such as logotherapy, was found to be protective against the development of physical disability. Retirement, which may lead to a reduced sense of purpose and meaning in life, was found to be a risk factor for this outcome.

### Caregiving in Veterans

Employment may play a role in fostering a greater sense of purpose in life, and with the aging veteran population reaching retirement age, understanding the roles that veterans maintain later in life could be a useful mechanism for promoting successful aging. Indeed, older veterans may transition to providing care to their family members as they reach retirement or face other challenges, as it is estimated that one in 10 of all caregivers in the U.S. have served in the military (63). Monin et al. (55) found that greater perceived resilience was negatively associated with veteran caregiver physical strain. Depressive symptoms were positively associated with emotional strain (55). Additionally, gratitude, happiness, and social support were associated with greater perceived rewards related to caregiving (55).

### Social Connectedness and Psychological Resilience

Social connectedness recurrently appears as an important factor in successful aging and resilience among older veterans who participated in the NHRVS. Fanning and Pietrzak (51) found that social connectedness was negatively related to SI in older veterans. Weiner et al. (60) investigated the effects of different types of social engagement in older veterans, and found that community integration, but not perceived social support, was associated with fewer mental health difficulties in older veterans. In a study of sexual minority veterans [i.e., lesbian, gay, bisexual; Monin et al. (61)] found that, relative to younger veterans, older veterans had lower levels of mental health problems, but they reported the smallest social support networks. Finally, Kuwert et al. (53) found that 44.0% of veterans reporting feeling lonely at least some of the time, with 10.4% reporting often feeling lonely. While depressive symptoms were strongly related to loneliness in this study, greater, perceived social support and having a attachment style were negatively related to this outcome.

### Psychological Resilience in Late-Life

Significant changes and losses are common in older adulthood and include retirement, erosion of social networks, and reductions in functioning and mobility. It has been proposed that experiencing trauma and stressors before late adulthood may “inoculate” and help enhance coping skills in older adults (52, 64). It has been hypothesized that experiencing trauma or stressors earlier in life may promote psychological resilience to future traumas and stressors (52, 64). Using data from the NHRVS, Pietrzak and Cook (52) found that among older veterans who endured a high number of traumas in their lifetimes, nearly 70% were psychologically resilient in later life. Relative to distressed veterans, resilient veterans were younger, more likely to be White, less likely to have physical health difficulties and psychiatric histories. Resilient veterans also endorsed higher levels of emotional stability, prosocial behaviors (e.g., altruism), gratitude, purpose in life, and lower levels of openness to experiences (52). These findings suggest that prevention and treatment efforts designed to enhance gratitude, sense of purpose, and altruism may help promote resilience in trauma-exposed veterans.

Negative age stereotypes, defined as deprecating beliefs about older people as a category, have been linked to a broad range of negative health outcomes, including cardiovascular disease, cognitive decline, and mortality (65). Using NHRVS data, Levy et al. (54) evaluated whether negative age stereotypes may also be linked to risk for mental disorders in older veterans who participated in the NHRVS. They found that the prevalence of SI (5.0 vs. 30.1%), anxiety (3.6 vs. 34.9%), and PTSD (2.0 vs. 18.5%) was significantly lower among older veterans who fully resisted negative age stereotypes, compared to those who fully accepted them. A 4-year prospective cohort study of this cohort further revealed that greater resistance of negative age stereotypes was linked to significantly lower incidence of these outcomes and that engagement in active coping moderated this association (62); specifically, among veterans with more negative age stereotypes, those who engaged in active coping strategies to manage stress were less likely to develop mental health problems relative to those who did not engage in these strategies. Collectively, these results suggest that strategies to promote positive age stereotypes (66) and engagement in active coping may help mitigate risk for mental illness in older veterans.

### Barriers to Mental Healthcare

Between 41 and 79% of older persons with psychiatric disorders do not receive mental health care (67, 68). Perceived barriers to care, including stigma, negative beliefs about mental health care, and logistical barriers to care, may also affect utilization of mental health services among older veterans (57). Blais et al. (57) analyzed NHRVS data to identify correlates of current mental health care utilization and perceived barriers to care in older veterans. Only 6% of older veterans reported current mental health care utilization, and among veterans who screened positive for a current psychiatric disorder 25% were currently utilizing services. Utilization was also associated with several medical and psychiatric disorders, most notably PTSD (odds ratio = 5.9). Notably, greater perceptions of stigma and negative beliefs about mental health care were related to decreased likelihood of utilizing care. Collectively, these results suggest that efforts to identify veterans with mental health distress, and to reduce stigma and negative beliefs about mental health care may help promote mental health service utilization among symptomatic older U.S. veterans.

### Clinical Implications

A majority of veterans with a high lifetime trauma burden are psychologically resilient in later life (52). However, a significant minority of older veterans may experience a clinically significant exacerbation of PTSD symptoms in late life (12). Prevention and treatment efforts designed to promote health behaviors, protective psychosocial characteristics (i.e., resilience, gratitude, purpose in life), social connectedness (i.e., secure attachment, community integration, social engagement), and cognitive functioning may help promote successful aging in older veterans and mitigate risk for mental disorders (12, 51–53, 56, 58, 60). Further, since physical disability is prevalent among older veterans, promoting a greater sense of purpose in life may help preserve physical functioning in aging veterans (59). Efforts to identify distressed older veterans and reduce stigma and negative beliefs about mental health care may help increase mental health service utilization (57). In sum, results of these studies suggest that interventions designed to mitigate psychological and physical struggles in older veterans, and to promote social connectedness and protective psychosocial characteristics, may help foster successful aging in veterans.

## Special Topics Relevant to Veterans

Twenty-five (see Table 4) studies from the NHRVS have examined a myriad of special topics relevant to veterans, including military sexual trauma, combat exposure, positive and negative effects of military service, homelessness, and psychiatric and physical morbidities/comorbidities.

**Table 4:** Special topics relevant to veterans.

**Citation**	**Title**	**Sample**	**Findings**	**Clinical implications**
Klingensmith et al. (69)	Military sexual trauma in US veterans: Results from the National Health and Resilience in Veterans Study	Cohort 2 (*n* = 1,468)	Almost 8% of US veterans screened positive for MST, with higher rates among female and younger veterans. MST was associated with elevated rates of comorbidities and suicidality, reduced functioning and quality of life.	Screening for MST may help identify veterans who are at risk for a broad range of mental health and functional difficulties, as well as increased need for mental health services.
Tsai et al. (70)	U.S. female veterans who do and do not rely on VA Health care: Needs and barriers to mental health treatment	Cohort 1 and Cohort 2 (*n* = 1,202)	Female VA patients were more likely to be low income, not employed, from racial-ethnic minority groups, combat veterans, and were more likely to have a disability, screen positive for PTSD, and report poorer mental health-related functioning than non-VA female veterans.	VA may serve as a health care “safety net” for many female veterans; female and male veterans experience similar stigma-related barriers to mental health care, such as fear of being perceived as weak.
Fuehrlein et al. (71)	The burden of alcohol use disorders in US military veterans: Results from the National Health and Resilience in Veterans Study	Cohort 1 (*n* = 3,157)	Younger age, male sex, lower education, lower income and greater number of life-time traumatic events were associated with life-time AUD.	Comprehensive assessment of trauma histories, as well as monitoring and treatment of trauma-related psychopathology and suicidality, may help mitigate risk for AUD in veterans.
Sippel et al. (72)	The burden of hostility in U.S. veterans: Results from the National Health and Resilience in Veterans Study	Cohort 1 Baseline and Wave 2 (*n* = 2,157)	Protective psychosocial characteristics and aspects of social connectedness were negatively associated with hostility. Psychological distress predicted all symptomatic hostility courses, while alcohol misuse predicted chronic aggressive urges and all symptomatic courses of difficulties controlling anger.	Aggressive urges and difficulties controlling anger are prevalent in U.S. veterans. Integrated treatments that target psychological distress and alcohol use problems, and bolster protective psychosocial factors and social connectedness may be helpful in mitigating hostility and anger in veterans.
Tsai et al. (73)	Homelessness among a nationally representative sample of US veterans: Prevalence, service utilization, and correlates	Cohort 1 Wave 3 (*n* = 1,533)	Among all veterans, 8.5% reported any lifetime homelessness in their adult life, but only 17.2% of those reported using VA homeless or social services.	Veterans with low income, and poor mental and physical health, were more likely to have experienced homelessness and may most benefit from targeted services. There may also be barriers to care for many homeless veterans, especially for those who live in rural areas.
Heinz et al. (74)	American military veteran entrepreneurs: A comprehensive profile of demographic, service history, and psychosocial characteristics	Cohort 1 (*n* = 1,285)	Self-employed veterans were more likely to serve in Vietnam and to serve in the military for fewer years. Higher levels of openness, extraversion, optimism, purpose in life, and greater need for autonomy and professional development were observed among self-employed veterans.	Results highlights the significant scope of job creation and economic activity driven by self-employed veterans; they also highlight the psychosocial profile of this population.
Stefanovics et al. (75)	Gambling in a national U.S. veteran population: Prevalence, sociodemographics, and psychiatric comorbidities	Cohort 1 (*n* = 3,157)	More than a third of U.S. veterans gamble recreationally, with a 2.2% screening positive for problem gambling. Both recreational and problem gambling were associated with elevated trauma burden and psychiatric comorbidities.	Findings underscore the potential utility of screening and treatment initiatives for gambling among veterans, and of the importance of trauma as a risk factor for problem gambling in veterans.
Thomas et al. (76)	Mental and physical health conditions in US combat veterans: Results from the National Health and Resilience in Veterans Study	Cohort 2 (*n* = 1,480)	Compared to non-combat veterans in the United States, combat veterans have elevated rates of PTSD, suicide attempt, stroke, and chronic pain independent of other sociodemographic, military, and mental health factors.	Identifying, screening, and treating combat veterans, and promoting access to and use of integrated mental and physical health services is extremely important, as combat veterans potentially face complex mental and physical health symptoms after deployment.
Ziobrowski et al. (77)	Gender differences in mental and physical health conditions in U.S. veterans: Results from the National Health and Resilience in Veterans Study	Cohort 1 (*n* = 3,157)	Female veterans had higher prevalence estimates of lifetime and current psychiatric conditions, and lifetime histories of arthritis, migraine headaches, and osteoporosis, but lower prevalence estimates of lifetime substance use disorders, and lifetime histories of diabetes, heart attack, and high blood pressure.	There are substantial gender differences in the prevalence of many health conditions in veterans. With more women joining the military, consideration of their unique health needs is critical for delivering gender-sensitive care and developing effective health interventions.
Campbell et al. (78)	Association between perceptions of military service and mental health problems in a nationally representative sample of United States military veterans	Cohort 2 (*n* = 1,484)	Desirable effects of service were more frequently endorsed than undesirable effects, but perceptions of military service were associated with suicidal ideation and other mental health conditions.	Findings underscore the importance of evaluating veterans' perceptions of service in therapy. Assessing perceptions of service could also be used as a method to identify possible at-risk veterans who might benefit from mental health services and resources.
Fuehrlein et al. (79)	Trajectories of alcohol consumption in U.S. military veterans: Results from the National Health and Resilience in Veterans Study	Cohort 1 Baseline, Wave 2, and Wave 3 (*n* = 3,150)	Lifetime major depressive disorder was linked to an excessive drinking trajectory, while fewer medical conditions and lower social support were linked to a moderate drinking trajectory. Having a secure attachment style and greater social support, and absence of lifetime MDD was linked to recovery from excessive drinking.	Targeting depression and related interpersonal factors such as attachment style and social support in population-based prevention and treatment initiatives may help prevent, mitigate, and promote recovery from excessive alcohol consumption in veterans.
Norman et al. (80)	The burden of co-occurring alcohol use disorder and PTSD in U.S. military veterans: Comorbidities, functioning, and suicidality	Cohort 1 (*n* = 3,157)	Veterans with AUD/PTSD were more likely to screen positive for depression, anxiety, suicidal ideation, to have attempted suicide. They also scored lower on measures of functioning and quality of life.	Findings highlight the clinical severity, including both mental and physical health burden, associated with having PTSD in addition to AUD and the importance of screening veterans for these disorders.
Stefanovics et al. (81)	The physical and mental health burden of obesity in U.S. veterans: Results from the National Health and Resilience in Veterans Study	Cohort 1 (*n* = 3,122)	Obesity was associated with greater trauma burden; elevated rates of a broad range of physical and mental health conditions, poor quality of life, and decreased engagement in an active lifestyle.	Results underscore the importance of multicomponent behavioral interventions and weight management interventions, and consideration of the role of trauma and PTSD as risk factors for obesity in veterans.
El-Gabalawy et al. (82)	Physical health conditions associated with full and subthreshold PTSD in U.S. military veterans: Results from the National Health and Resilience in Veterans Study	Cohort 1 (*n* = 3,157)	Both full and subthreshold PTSD were associated with increased odds of sleep disorder and respiratory conditions. Full PTSD was associated with increased odds of osteoporosis or osteopenia and migraine, while subthreshold PTSD only was associated with increased odds of diabetes.	Dysphoric arousal symptoms of PTSD are particularly strongly linked to physical conditions. Sleep disturbances, in particular, may drive the relationship between PTSD symptoms, and respiratory and inflammatory diseases, and may be targeted in integrated mental-physical health interventions.
Straus et al. (83)	Differences in protective factors among U.S. Veterans with post-traumatic stress disorder, alcohol use disorder, and their comorbidity: Results from the National Health and Resilience in Veterans Study	Cohort 1 (*n* = 3,157)	Comorbid PTSD/AUD had lower social/psychosocial protective factors than AUD-only. Social/psychosocial factors partially mediated PTSD and suicidal ideation. Only psychosocial factors partially mediated link between PTSD and suicide attempts.	Enhancing protective factors within psychological treatments, such as strength-based interventions that aim to foster pre-existing protective factors, may contribute to better clinical outcomes in veterans with PTSD and AUD.
Meffert et al. (2)	US veterans who do and do not utilize veterans affairs health care services: Demographic, military, medical, and psychosocial characteristics	Cohort 1 (*n* = 3,152)	Veterans who use VA services have a substantially elevated mental and physical health burden compared to other veterans. The primary factor differentiating VA users from those that did not was presence of lifetime psychopathology.	Results highlight the importance of specialty VA services targeting PTSD, depression, anxiety, social phobia, DUD, and suicidality. Education efforts targeting cultural and/or social norms that interfere with help-seeking behavior in veterans may also help connect veterans to care.
Nichter et al. (84)	Psychological burden of PTSD, depression, and their comorbidity in the U.S. veteran population: Suicidality, functioning, and service utilization	Cohort 1 (*n* = 2,732)	Veterans with probable PTSD/MDD were more likely to have attempted suicide and scored lower on measures of functioning and quality of life and screen positive for current suicidal ideation, lifetime suicide attempts, probable generalized anxiety and social anxiety disorders.	Results suggest that the deleterious effects of PTSD and MDD on mental health functioning and QoL are additive, such that their co-occurrence is associated with the largest magnitude of impairment relative to each disorder alone.
Nichter et al. (85)	Physical health burden of PTSD, depression, and their comorbidity in the U.S. veteran population: Morbidity, functioning, and disability	Cohort 1 (*n* = 2,732)	Veterans with comorbid PTSD/MDD were more likely to be diagnosed with heart disease, migraine, fibromyalgia, and rheumatoid arthritis compared to those with MDD-only, and were at greater odds of being diagnosed with hypercholesterolemia and hypertension relative to those with PTSD-only.	Veterans with co-occurring PTSD/MDD may be at increased risk for the development of cardiovascular disease. Results suggest the importance of integrating mental health services in primary care settings to increase access and utilization among older veterans.
Averill et al. (86)	Sex differences in correlates of risk and resilience associated with military sexual trauma	Cohort 2 (*n* = 115)	Compared to female MST survivors, male MST survivors reported increased lifetime traumatic events, hostility, and history of drug use disorder, whereas female veterans reported increased lifetime PTSD symptoms.	Results suggest that there may be gender-specific clinical profiles in veterans who experienced MST, which may help inform personalized interventions in this population.
Baldassarri et al. (87)	Nicotine dependence in US military veterans: Results from the National Health and Resilience in Veterans Study	Cohort 1 (*n* = 3,157)	19.4% of veterans met criteria for lifetime nicotine dependence. Veterans with lifetime nicotine dependence were more likely to have psychiatric and medical conditions and lower physical functioning compared with veterans without.	Veterans with lifetime nicotine dependence may require a comprehensive and integrated approach to mental and physical health care, which includes attention to mental and physical health comorbidities.
Carr et al. (88)	Race, ethnicity, and clinical features of alcohol use disorder among US military veterans: Results from the National Health and Resilience in Veterans Study	Cohort 1 (*n* = 1,212)	Black and Hispanic veterans with lifetime AUD may experience a higher disease burden relative to white veterans.	Results suggest higher disease burden for racial/ethnic minority veterans with AUD and underscore the importance of race/ethnicity-sensitive assessment, monitoring, and treatment of AUD for veterans.
Nitcher et al. (89)	Risk and protective factors associated with comorbid PTSD and depression in U.S. military veterans: Results from the National Health and Resilience in Veterans Study	Cohort 1 (*n* = 2,732)	Racial/ethnic minority status, number of lifetime traumas, and time spent engaged in private religious/spiritual activities were associated with PTSD/MDD status, while higher scores on measures of community integration and dispositional optimism were negatively associated with comorbid PTSD/MDD status.	Results underscore the need to study whether targeting dispositional optimism and community integration in prevention and treatment efforts for PTSD/MDD may enhance clinical outcomes in this difficult-to-treat population.
Straus et al. (90)	Determinants of new-onset alcohol use disorder in U.S. military veterans: Results from the National Health and Resilience in Veterans Study	Cohort 1 Baseline, Wave 2, Wave 3, and Wave 4 (*n* = 1,770)	Approximately 6% of veterans developed AUD over 7-year follow-up period. Drug disorders, alcohol use, and trauma characteristics were associated with risk of developing AUD.	Results underscore the importance of prior substance use history, as well as trauma-related factors such as adult sexual trauma and anxious arousal symptoms, as determinants of incident AUD.
Stefanovics et al. (91)	PTSD and obesity in U.S. military veterans: Prevalence, health burden, and suicidality.	Cohort 1 (*n* = 3,157)	5.8% of U.S. veterans have co-occurring PTSD and obesity. Veterans with PTSD and obesity have elevated mental and physical health burden and suicide risk.	Integrated, multi-component interventions targeting PTSD and comorbid obesity may help mitigate the elevated mental and physical health burden in this population.
Stefanovics et al. (92)	Smoking, obesity, and their co-occurrence in the U.S. military veterans: Results from the National Health and Resilience in Veterans Study	Cohort 1 (*n* = 3,157)	The prevalence of co-occurring obesity and smoking among U.S. veterans was 5.4%. Co-occurring obesity and smoking was positively associated with mental and physical health problems, higher level of trauma and stress burden, and lower quality of life.	Findings underscore the importance of multi-component interventions targeting obesity and smoking in veterans.

### Military Sexual Trauma and Combat Exposure

Certain trauma exposures are unique to veterans, including military sexual trauma and combat exposure. While the prevalence of MST is highest in female veterans, the VA reports that almost half of VA users who screen positive for MST are men (93). Furthermore, MST is also thought to be largely underreported, particularly in men, among whom there may be a higher burden of stigma. Using NHRVS data, Klingensmith et al. (69) found that 7.6% of U.S. military veterans reported MST, and that the prevalence was significantly higher among female than male veterans (32.4 vs. 4.8%) and younger than older veterans (22.8% among 18–29 year-olds vs. 4.5% among 60+ year-olds). In a model adjusted for sociodemographic and military characteristics, MST was associated with elevated rates of several psychiatric morbidities and suicidality (adjusted odds ratio range = 2.2–3.1), reduced functioning and QOL, as well as increased mental health treatment utilization (adjusted odds ratios range = 2.4–3.7) (69). A follow-up study by Averill et al. (86) found that, relative to female MST survivors, male MST survivors have greater trauma burden, hostility, and higher rates of drug use disorder, but lower severity of PTSD symptoms. Taken together, these findings suggest that screening for MST and the consideration of sex differences are critical to informing risk for a broad range of mental health problems in U.S. veterans.

Similar to MST, combat exposure is linked to increased risk for mental health problems, including psychiatric disorders such as PTSD, generalized anxiety disorder, MDD, and substance use (94, 95). A NHRVS study (76) found 38% of veterans reported being exposed to combat, and that, relative to non-combat veterans, combat veterans had 2 to 3-fold elevated rates of PTSD and generalized anxiety disorder. Further, combat veterans had 68% greater odds of a suicide attempt and 85% and 38% greater odds of a stroke and chronic pain, respectively. Among combat veterans, age was associated with differential risk for certain health conditions, with younger veterans more likely to screen positive for PTSD, SI, and migraine headaches, while older veterans were more likely to reported having heart disease and a heart attack. Results of this study suggest that combat exposure may independently contribute to risk for mental and physical health issues in U.S. veterans, and that age may moderate the effect of combat exposure on health outcomes.

### Perceived Effects of Military Service

In addition to combat exposure, other military-related factors, such as perceived threat during deployment, and difficult living and working environments, have been linked to depression, anxiety, and PTSD (96). Using NHRVS data, Campbell et al. (78) examined the relationship between perceptions of desirable (e.g., military service helped one learn to cope with adversity) and undesirable effects of service (e.g., military service caused misery and discomfort), and mental health problems. The study found desirable effects of service were more frequently endorsed than undesirable effects (54–86% vs. 9–48%), and that combat-exposed veterans were more likely to endorse undesirable than desirable effects of service (11–60% vs. 4–41%). Of note, after adjustment for possible confounding variables, undesirable effects of service predicted significantly greater odds of probable current mental health disorders and current SI (both odds ratios = 1.1), while desirable effects of military service were linked to lower odds of current SI (odds ratio = 0.96). Taken together, results of this study suggest that perceptions of military service may be linked to risk for mental disorders and suicidality, and that desirable effects of effects of military service may help counteract risk for suicidal thinking associated with undesirable effects of service. Clinically, they suggest that assessment of perceptions of military service may help identify at-risk veterans who may benefit from mental health treatment.

### Major Depressive Disorder

MDD is prevalent disorder that often co-occurs with PTSD. Using data from the NHRVS, Nichter et al. (84) estimated the prevalence of current PTSD/MDD in the U.S. veterans population at 3.4%. Compared to veterans with PTSD only and MDD only, those with comorbid PTSD/MDD were significantly more likely to screen positive for current SI, lifetime suicide attempts, and anxiety disorders, and they scored substantially lower on measures of mental health and cognitive functioning, and QOL. Furthermore, a follow-up study (85) found that veterans with comorbid PTSD/MDD had a substantially greater burden of physical illness than veterans with either disorder alone. Specifically, veterans with comorbid PTSD/MDD had higher rates of heart disease, migraine, fibromyalgia, and rheumatoid arthritis compared to veterans with MDD alone, and higher rates of hypercholesterolemia and hypertension compare to veterans with PTSD alone (85). Taken together, these findings highlight the importance of screening, monitoring, and treatment comorbid PTSD/MDD in veterans. They also suggest that veterans with comorbid PTSD/MDD should be closely monitored for physical health problems, particularly cardiovascular risk factors and disease, and inflammatory and pain-related conditions. Finally, emerging NHRVS research on PTSD/MDD comorbidity (89) has revealed that greater dispositional optimism and community integration were associated with lower likelihood of having comorbid PTSD/MDD relative to either disorder alone, thus highlighting the potential importance of targeting these psychosocial factors in prevention and treatment efforts.

### Alcohol Use Disorder

Although PTSD is one of the most prevalent and intensively studied psychiatric disorders among veterans, other disorders are also prevalent among veterans. With regard to alcohol use disorder (AUD), Fuehrlein et al. (71) found the lifetime and past-year prevalence of AUD was 42.2 and 14.8%, respectively in the NHRVS. Results further revealed that veterans with lifetime AUD were approximately four times more likely to have a lifetime history of PTSD, MDD, and SI (78). When considering racial/ethnic differences in veterans associated with AUD, a recent study found that Black and Hispanic veterans with lifetime AUD experience a greater disease burden relative to White veterans, which underscores the importance of race/ethnicity-sensitive approaches to the assessment, monitoring, and treatment of AUD in veterans (88).

Building on these cross-sectional findings of AUD in veterans, a 4-year prospective cohort study (79) was conducted to identify predominant trajectories of alcohol consumption and baseline determinants of these trajectories, where four predominant trajectories were identified. The majority (65.3%) of veterans were rare drinkers, 30.2% were moderate drinkers, 2.6% were excessive drinkers (2.6%), and 1.9% were recovering drinkers. Lifetime MDD was linked to an excessive drinking trajectory, while fewer medical conditions and lower social support were linked to a moderate drinking trajectory. Absence of lifetime MDD, having a secure attachment style, and greater social support were linked to the recovering drinking trajectory (84). Another prospective study (90) found that ~6% of veterans without AUD at baseline developed AUD over 7-year follow-up. Adult sexual trauma in adulthood, higher anxious arousal symptoms of PTSD, lifetime history of drug and nicotine use disorders, and higher alcohol consumption at baseline predicted the development of AUD. Collectively, these results suggest that targeting MDD, other substance use, and trauma exposure in population-based prevention and treatment initiatives may help prevent, mitigate, and promote recovery from AUD in veterans.

Given that AUD and PTSD are among the most prevalent disorders in veterans and often co-occur, it is important to determine the burden associated with AUD/PTSD comorbidity relative to either disorder alone. Using NHRVS data, Norman et al. (80) found that one of every five veterans with AUD also screened positive for PTSD. Veterans with comorbid PTSD/AUD, comparted to AUD only veterans, were more likely to screen positive for MDD, GAD, and reported strikingly higher rates of current SI (39.1 vs. 7.0%) and lifetime suicide attempt(s) (46.0 vs. 4.1%); they also scored lower on measures of cognitive, mental, and physical health functioning, and QOL (80). Building on this study, Straus et al. (83) examined social (e.g., social connectedness) and psychosocial characteristics in veterans with PTSD, AUD, and comorbid PTSD/AUD. The study found veterans with comorbid PTSD/AUD had lower on social connectedness and protective psychosocial characteristics relative to those with AUD alone, but not PTSD alone. While both social and psychosocial protective factors partially mediated the relation between PTSD and current SI, only psychosocial protective characteristics partially mediated the relation between PTSD and lifetime suicide attempt(s) (83). Collectively, these findings highlight the burden of comorbid PTSD/AUD in veterans and suggest that treatment of PTSD in veterans with PTSD/AUD, and promotion of social connectedness and psychosocial protective factors, may help mitigate risk for and promote recovery from these disorders.

### Nicotine Dependence

Another commonly used substance in veterans is nicotine. Baldassarri et al. (87) found that almost one in five U.S. veterans met criteria for lifetime nicotine dependence. The strongest correlates of lifetime nicotine dependence were lifetime alcohol use disorder, lifetime drug use disorder, current alcohol use disorder, kidney disease, and heart disease. Given that nicotine dependence often presents as part of a complex set of conditions that includes psychiatric and medical comorbidities, trauma history, reduced overall physical functioning, and an increase in somatic complaints, veterans with nicotine dependence may require a comprehensive and integrated approach to care.

### Problem Gambling

PTSD has also been associated with problem gambling (97) and 40% of veterans seeking treatment for gambling problems have reported prior suicide attempts, with 64% of those who attempted suicide reported gambling-related attempts (98). Using NHRVS data, Stefanovics et al. (75) examined the prevalence, risk factors, and mental health correlates of recreational and problem gambling in U.S. veterans. They found 35.1% of U.S. veterans gambled recreationally and 2.2% screened positive for problem gambling. Younger age, self-identifying as Black and being retired were associated with increased likelihood of screening positive for problem gambling. Veterans with problem gambling also had higher rates of substance use, anxiety, depressive disorders, a history of physical trauma or sexual trauma, and greater lifetime trauma burden. Results of this study suggest that a significant minority of U.S. veterans screen positive for problem gambling, which is associated with greater mental health burden. They further suggest that routine screening and monitoring of gambling severity may help identify at-risk veterans, and that trauma burden may contribute to risk for problem gambling in this population.

### Physical Health Morbidities

The relationship between trauma, PTSD, and physical health has been well-documented (99). Given that the clinical presentation of PTSD is often heterogeneous, examining the relation between PTSD and subthreshold PTSD, and a range of physical conditions may elucidate potential mechanisms driving comorbidity with physical health conditions. Using data from the NHRVS, El-Gabalawy et al. (82) found that PTSD and subthreshold PTSD were associated with increased risk of sleep disorder and respiratory conditions. PTSD was additionally associated with increased risk of osteoporosis or osteopenia and migraine, while subthreshold PTSD was associated with increased odds of diabetes. Results also demonstrated the importance of dysphoric arousal symptoms of PTSD, which are characterized by sleep disturbance, concentration difficulties, and irritability and anger, in risk models of certain physical conditions in veterans with PTSD symptoms.

NHRVS investigators have also examined the prevalence and health burden of obesity in U.S. veterans. A recent study found that 32.7% of NHRVS veterans were obese, which is higher than previously reported estimates in the U.S. veteran population (81). The prevalence of obesity was particularly elevated among veterans who were younger, racial/ethnic minorities, and who utilized VA healthcare services as their main source of healthcare. Notably, obesity was associated with greater trauma burden, as well as elevated rates of a broad range of mental health conditions, including PTSD and nicotine dependence (81). Further NHRVS studies have revealed that 5.8% of veterans have co-occurring PTSD and obesity and 5.4% of veterans have co-occurring nicotine dependence and obesity (91, 92). Obesity was also associated with a range of physical health conditions, such as diabetes, arthritis, and heart disease, in addition to poor physical and mental health-related functioning and overall QOL (91). Collectively, these findings underscore the burden of obesity—independently and in combination with smoking and PTSD—on multiple aspects of health, functioning, and QOL in veterans.

### Gender Differences

Gender differences have also been reported among veterans for specific health conditions. Risk for exposure to various types of traumatic events differs by gender (6) and the association of trauma with adverse health outcomes may vary by traumatic event type (69). Thus, it is important to consider the possibility that assaultive trauma may be differentially associated with health outcomes in male and female veterans. A recent study (77) found that female veterans had significantly higher prevalence of lifetime PTSD, MDD, arthritis, migraine headaches, and osteoporosis, but lower prevalence estimates of lifetime nicotine dependence, drug use, diabetes, heart attack, and high blood pressure. With more women joining the military, consideration of their unique health needs is critical to informing care delivery models and developing gender-sensitive interventions (77).

### Hostility and Anger

Understanding the burden and clinical features of hostility and anger is particularly relevant to veterans given evidence of elevated rates of hostility-related health issues such as PTSD, depression, and heart disease relative to non-veterans (100, 101). Using data from the NHRVS, Sippel et al. (72) examined the prevalence and longitudinal course of hostility over a 2-year period. They found that 61.2% of veterans reported experiencing difficulties controlling anger and that nearly a fourth reported having aggressive urges (72). Psychological distress and alcohol misuse were associated with symptomatic courses of hostility, while greater dispositional optimism and a secure attachment style were negatively associated with these courses (72). These findings underscore the burden of hostility and anger in the U.S. veterans, and suggest potential targets for prevention and treatment efforts designed to mitigate hostility and anger in this population.

### Homelessness

Studies from the NHRVS have also contributed to the literature on homelessness and employment, which may help inform allocation of governmental resources and services for veterans. A 2016 study by Tsai et al. (73) found that 8.5% of veterans reported ever being homeless in their adult life, but only 17.2% of those reported ever using VA homeless services. Findings further revealed that low income, being middle-aged (15, 18, 37–41, 45–47), and having poor mental and physical health were independently associated with lifetime homelessness. Additionally, veterans who were White or lived in rural areas were significantly less likely to have used ever VA homeless services.

### Self-Employment

Efforts to support self-employment may help mitigate unemployment among veterans. Results from Heinz et al. (74) demonstrated that veteran entrepreneurs experienced a higher number of traumas compared to non-entrepreneurs, but veterans entrepreneurs did not report higher levels of PTSD or other psychopathology. These results suggest that higher trait levels of optimism, extraversion, gratitude, curiosity (i.e., need for autonomy), and openness may contribute to resilience in veteran entrepreneurs. Combined with elevated sense of purpose in life, there trains may help these individuals be more “gritty” and pursue entrepreneurial employment.

### Clinical Implications

Veterans face a wide array of mental and physical health struggles, many of which commonly co-occur, and may result in functional difficulties, and chronicity and exacerbation of symptoms. Results of the NHRVS studies reviewed above help to characterize the population-based burden of a wide range of mental and physical health conditions that are prevalent among veterans, which may help inform outreach efforts, resource allocation, and program development within VA and non-VA settings to better serve this population. They also highlight the need for screening initiatives and specialty services targeting homelessness, employment, and PTSD and co-occurring health disorders. Specifically, veterans with histories of MST and combat-exposure, as well as common co-occurring physical and mental health conditions may have heightened need for screening, monitoring, and treatment efforts. Increasing access to information about mental health care, which may serve to decrease stigma, may also help veterans navigate barriers to initiation and engagement in care.

## Resilience and Post-Traumatic Growth

Although most studies on trauma focus on psychopathology and other negative consequences, a new concentration in trauma literature is to characterize the prevalence and correlates of psychological resilience in veterans. Psychological resilience is defined as “the ability to adapt in the aftermath of trauma or extreme stress and maintain a high level of psychological functioning” (3). There are several personality and behavioral constructs associated with stress resilience, including hardiness, mental toughness, and grit (102). Though these constructs have nuanced differences, they represent the positive psychological traits that may help foster psychological resilience. Additionally, positive psychological changes, or PTG, can occur as a consequence of exposure to traumatic and stressful life events, and may include developing an increased appreciation of life, greater sense of personal strength, renewed appreciation for intimate relationships, and positive spiritual changes (103). Longitudinal studies of resilience and PTG can help elucidate the nature and determinants of heterogeneous courses of reactions to stressful or traumatic events and help inform strategies for promoting positive psychological changes in the face of adversity (104). To date, eight NHRVS studies (see Table 5) have focused on resilience and post-traumatic growth.

**Table 5:** Resilience and post-traumatic growth.

**Citation**	**Title**	**Sample**	**Findings**	**Clinical implications**
Tsai et al. (105)	Post-traumatic growth among veterans in the USA: Results from the National Health and Resilience in Veterans Study	Cohort 1 (*n* = 3,157)	50.1% of all veterans and 72.0% of veterans who screened positive for PTSD reported at least “moderate” PTG in relation to their worst traumatic event. A quadratic relationship was found to best explain the relationship between PTSD symptoms and PTG.	Greater social support, purpose in life and intrinsic religiosity were independently associated with PTG, suggesting that clinical interventions designed to promote these factors may help foster post-traumatic growth.
Tsai et al. (106)	What doesn't kill you makes you stronger: A national study of U.S. military veterans	Cohort 1 Baseline and Wave 2 (*n* = 1,057)	Greater scores on the Personal Strength domain of the PTG Inventory-Short Form at baseline was associated with reduced severity and incidence of PTSD at a 2-year follow-up.	Veterans who perceive greater gains in personal strength, perhaps as a result of developing greater coping skills in response to previous traumas, may be better able to cope with subsequent traumas.
Tsai et al. (104)	Longitudinal course of post-traumatic growth among U.S. military veterans: Results from the National Health and Resilience in Veterans Study	Cohort 1 Baseline and Wave 2 (*n* = 1,838)	Five courses of PTG were identified. More than half of veterans who reported at least “moderate” PTG maintained that level 2 years later. PTSD symptoms, medical conditions, purpose in life, altruism, gratitude, religiosity, and an active reading lifestyle predicted maintenance or increase in PTG.	Potentially reading and writing about the trauma may facilitate PTG in veterans with PTSD and physical health conditions may help reduce burdens from these disorders.
Isaacs et al. (107)	Psychological resilience in U.S. military veterans: A 2-year, nationally representative prospective cohort study	Cohort 1 Baseline and Wave 2 (*n* = 2,157)	Results suggest that the majority of trauma-exposed veterans (67.7%) are psychologically resilient. Higher levels of emotional stability, extraversion, gratitude, purpose in life, and altruism, and lower levels of openness to experiences predicted resilient status.	Purpose in life, gratitude, and altruism, are potentially modifiable, and thus suggest possible population-based targets for prevention and treatment efforts designed to bolster psychological resilience in veterans.
Sharma et al. (108)	Religion, spirituality, and mental health of U.S. military veterans: Results from the National Health and Resilience in Veterans Study	Cohort 1 (*n* = 3,151)	Higher levels of religion/spirituality were also strongly linked with increased dispositional gratitude, purpose in life, and post-traumatic growth.	Broader spiritual activities/practices, such as mindfulness, yoga, and breathing-based meditation may have beneficial effects in mitigating symptoms of PTSD, depression and anxiety.
Tsai and Pietrzak (109)	Trajectories of post-traumatic growth among US military veterans: A 4-year nationally representative, prospective cohort study	Cohort 1 Baseline, Wave 2, and Wave 3 (*n* = 2,718)	Greater severity of re-experiencing and avoidance PTSD symptoms at baseline predicted “Consistently Moderate” and “High and Increasing” PTG trajectories. Compared to the “Low and Decreasing” trajectory, the “High and Increasing PTG” trajectory scored higher on baseline measures of gratitude, purpose in life, spirituality, and social support.	Multidisciplinary approaches that include peer support programs, spiritual leaders, and loved ones in the clinical process may be needed to enhance these deep philosophical domains of life and functioning, and help facilitate PTG.
Martz et al. (110)	Post-traumatic growth moderates the effect of post-traumatic stress on quality of life in U.S. military veterans with life-threatening illness or injury	Cohort 1 (*n* = 418)	PTSD was inversely associated with quality of life. Both PTGI-Total and all five PTGI subscales successfully moderated the influence of PTSD on perceived quality of life.	Acceptance and Commitment Therapy or Coping Effectiveness Training may help individuals with life-threatening injuries and medical conditions with finding some positive aspects of life that can instill new meanings, despite the fact that their medical conditions may never be cured and could even worsen.
Whealin et al. (111)	Dynamic interplay between PTSD symptoms and post-traumatic growth in older military veterans	Cohort 1 Baseline, Wave 2, and Wave 3 (*n* = 2,006)	Post-traumatic stress disorder symptoms had strong associations with both current and subsequent post-traumatic growth, with the relationship optimally characterized by a non-linear, “inverted U” shaped association in which moderate PTSD symptoms are associated with the greatest gains in PTG over time.	Interventions to promote deliberate, constructive attempts to manage chronic PTSD symptoms via active and religious coping may help promote greater post-traumatic growth in trauma-exposed veterans

### Psychological Resilience

As described above, studies from the NHRVS have found that the majority of veterans with a large number of traumatic experiences are psychologically resilient in later life and that prosocial behaviors and purpose in life may help promote psychological resilience (52). Although many cross-sectional studies have examined the correlates of veteran resilience, scarce longitudinal studies have identified longitudinal determinants of resilience in this population. Longitudinal data are important, as they can help inform population-based treatment and prevention initiatives geared toward the promotion of psychological resilience in trauma-exposed individuals such as veterans.

Toward this end, Isaacs et al. (107) conducted a 2-year prospective cohort study and found that among veterans endured a high number of traumas over the course of their lifetimes, 67.7% reported minimal-to-no current psychological distress (i.e., current PTSD, depression, and anxiety symptoms). Baseline determinants of resilience included younger age, White race/ethnicity, better physical health, lower rates of psychiatric and substance use disorders, and greater levels of emotional stability, extraversion, purpose in life, dispositional gratitude, and altruism, and lower openness to experiences (107). Cross-sectionally, research using the NHRVS has also found high levels of religiosity/spirituality is associated with decreased risk for PTSD, MDD, alcohol use disorder, and SI (108). Importantly, higher levels of religiosity/spirituality were also strongly linked with greater PTG and other protective factors, such as increased purpose in life and dispositional gratitude, thus underscoring the potential importance of religiosity/spirituality in contributing to psychological resilience in U.S. veterans.

### Post-traumatic Growth

A growing body of literature has found that individuals who experience a wide range of traumatic life events (e.g., prisoners of war, refugees, assault survivors, combat veterans) often report experiencing PTG. However, the relationship between PTSD symptoms and PTG is less clear. In the first known nationally representative study of PTG in veterans, Tsai et al. (105) found that nearly three-quarters of veterans who screened positive for PTSD reported at least moderate levels of PTG. Several psychosocial factors, such as greater social connectedness, intrinsic religiosity and purpose in life, were also independently related to greater PTG (105). Furthermore, they observed a curvilinear (i.e., inverted U-shaped) relationship between PTSD symptoms and PTG, with veterans with a moderate level of PTSD symptoms reporting the greatest levels of PTG. A follow-up prospective study (106) examined whether PTG may predict greater resilience to subsequent traumatic stress. Results indicated that greater scores on the personal strength domain of PTG, which assesses one's perception of their ability to handle difficulties, was associated with reduced severity and incidence of PTSD at a 2-year follow-up (106). Other research on PTG using NHRVS data has found that greater PTG moderates the influence of PTSD on perceived QOL in veterans with life-threatening illness or injury, with higher levels of PTG associated with higher QOL among veterans with greater severity of PTSD symptoms (110).

To date, the vast majority of studies of PTG have been cross-sectional in nature, thus little is known about the longitudinal course or predictors of PTG. Consistent with the cross-sectional studies, a recent prospective study (111) of the dynamic interplay between PTSD symptoms and perceived PTG found that the relationship between PTSD and PTG over time was optimally characterized by a non-linear, “inverted U” shaped association, and that greater severity of PTSD symptoms, particularly avoidance and hyperarousal, were associated with greater PTG over time, but not vice versa. Another study of PTG in the NHRVS found that over a 2-year period, PTSD symptoms, particularly re-experiencing symptoms, greater number of medical conditions, stronger purpose in life, altruism, and an active lifestyle predicted a maintenance or increase in PTG over time (104). Using 4-year prospective data, Tsai and Pietrzak (109) identified three predominant PTG trajectories (i.e., low and decreasing, consistently moderate, and high and increasing) among veterans in hopes of better understanding the temporal course of PTG. Veterans who reported experiencing greater severity of PTSD symptoms, particularly re-experiencing and avoidance symptoms, were more likely to have consistently moderate or high and increasing PTG (109).

### Clinical Implications

In clinical settings, individuals with trauma-exposure generally receive treatments designed to reduce negative symptoms; however, a growing body of research suggests the potential importance of additionally considering interventions designed to foster resilience and PTG (110). Specifically, prevention and treatment efforts designed to enhance modifiable factors such as sense of purpose and meaning in life, dispositional gratitude, and altruism may help promote resilience and PTG in trauma-exposed veterans (104, 107, 109). Further, promoting positive health behaviors (e.g., regular physical activity), and screening and treating medical and mental health conditions may also help bolster psychological resilience and PTG. Furthermore, since re-traumatization is common in trauma-exposed individuals, fostering PTG in clinical settings may help promote psychological resilience in response to subsequent traumatic life events.

## Genetics and Epigenetics

Many major psychiatric disorders have high heritability. To date, twelve studies (see Table 6) have investigated genetic factors associated with major psychiatric disorders such as PTSD and MDD in the NHRVS sample. These studies provide preliminary insight into how genetic factors may increase risk for lifetime PTSD and related disorders in European-American veterans, as well as how environmental factors such as trauma burden and social support may exacerbate or moderate risk for these disorders. Of note, however, results of these studies should be interpreted with caution for the following reasons: first, they primarily focused on candidate genes previously found to be associated with PTSD and related disorders that have not emerged as statistically significant in recent genome-wide association studies; second, they included only European-Americans; and third, they were based on relatively small samples, which were underpowered for genome-wide association studies.

**Table 6:** Genetics and epigenetics.

**Citation**	**Title**	**Sample**	**Findings**	**Clinical implications**
Pietrzak et al. (112)	Association between negative age stereotypes and accelerated cellular aging: Evidence from two cohorts of older adults	Cohort 1 (*n* = 335)	Negative age stereotypes predicted shorter telomere length in veterans age 60 and older.	Since negative age stereotypes can be modified, such interventions may help prevent premature cellular aging as well as age-related functional decline.
Watkins et al. (113)	FKBP5 polymorphisms, childhood abuse, and PTSD symptoms: Results from the National Health and Resilience in Veterans Study	Cohort 1 (*n* = 1, 585) and Cohort 2 (*n* = 577)	*FKBP5* polymorphisms and childhood abuse may contribute to vulnerability for PTSD symptoms and may be most strongly associated with trauma-related hyperarousal symptoms that comprise this phenotype.	Findings may be useful in informing etiologic models of PTSD, by linking these candidate *FKBP5* polymorphisms to specific PTSD symptom clusters, most notably hyperarousal symptoms
Watkins et al. (114)	Hostility and telomere shortening among U.S. military veterans: Results from the National Health and Resilience in Veterans Study	Cohort 1 (*n* = 484)	Hostility, specifically, difficulties controlling anger, was associated with peripheral telomere shortening.	Results underscore the importance of assessing anger and aggression among veterans with high levels of hostility. Cognitive behavioral anger management treatments may be helpful in mitigating elevated hostility and associated acceleration of biological aging.
Andersen et al. (115)	Polygenic scores for major depressive disorder and risk of alcohol dependence	Cohort 1 (*n* = 2,036)	Higher major depression polygenic risk scores were associated with a significantly increased risk of alcohol dependence.	Findings suggest that common genetic factors contribute to MDD-AD comorbidity and that some individuals carry a genetic predisposition for both disorders.
Sippel et al. (116)	Oxytocin receptor gene polymorphisms, attachment, and PTSD: Results from the National Health and Resilience in Veterans Study	Cohort 1 (*n* = 1,657) and Cohort 2 (*n* = 506)	Insecure attachment style and the interaction of *OXTR* rs53576 and attachment style were associated with probable lifetime PTSD	Findings may inform biosocial models of PTSD and suggest that individuals with one or more *OXTR* rs53576 alleles may benefit from interventions that target the oxytocin system and efforts to improve attitudes and feelings toward relationships to facilitate social coping.
Watkins et al. (117)	Association between functional polymorphism in neuropeptide Y gene promoter rs16147 and resilience to traumatic stress in US military veterans	Cohort 1 (*n* = 1,585) and Cohort 2 (*n* = 557)	The T allele of *NPY* gene promoter rs16147 was associated with resiliency to cumulative traumatic stress, especially resilience to intrusive symptoms.	*NPY* rs16147 T allele carriers may be less physiologically reactive to trauma cues, because they produce greater levels of NPY during stress. Further research is needed to evaluate whether interventions designed to enhance NPY levels1 may help promote stress resilience in trauma-affected populations.
Mota et al. (118)	Apolipoprotein E gene polymorphism, trauma burden, and post-traumatic stress symptoms in U.S. military veterans: Results from the National Health and Resilience in Veterans Study	Cohort 1 (*n* = 1,386) and Cohort 2 (*n* = 509)	The interaction of *APOE* epsilon4 carrier status and cumulative trauma burden was associated with greater severity of PTSD symptoms. Greater social support was associated with lower severity of PTSD symptoms among *APOE* epsilon4 allele carriers with greater cumulative trauma burden.	Enhancement of social support networks among highly trauma-exposed veterans at elevated genetic risk for PTSD may help mitigate PTSD symptoms
Averill et al. (119)	Apolipoprotein E gene polymorphism, post-traumatic stress disorder, and cognitive function in older U.S. veterans: Results from the National Health and Resilience in Veterans Study	Cohort 1 (*n* = 1,386) and Cohort 2 (*n* = 509)	APOE epsilon4 allele carriers with PTSD had substantially greater cognitive difficulties than epsilon4 carriers without PTSD.	Findings underscore the importance of assessing, monitoring, and treating PTSD in veterans and other trauma-affected populations who are at increased genetic risk for cognitive decline and dementia.
Pitts et al. (120)	BDNF Val66Met polymorphism and post-traumatic stress symptoms in U.S. military veterans: Protective effect of physical exercise	Cohort 1 (*n* = 1,386) and Cohort 2 (*n* = 509)	Met allele carriers reported greater severity of lifetime and current PTSD symptoms, specifically re-experiencing symptoms, with a significant interaction between Met carrier status and lifetime trauma load. Exercise moderated the interaction.	Findings suggest that interventions designed to bolster engagement in physical exercise may help mitigate PTSD symptoms in veterans who are Met allele carriers and highly exposed to trauma.
Tamman et al. (121)	Accelerated DNA methylation aging in U.S. military veterans: Results from the National Health and Resilience in Veterans Study	Cohort 1 (*n* = 1,135)	Psychosocial factors of lifetime trauma burden, child sexual trauma, and negative beliefs about aging were independently associated with DNA aging. Diabetes, hypertension, and body mass index also emerged as correlates of DNA aging.	Findings underscore the importance of interventions targeting negative beliefs about aging and modifiable factors linked to DNA aging in veterans at increased risk of age-related morbidities and mortality.
Tamman et al. (122)	Attachment style moderates effects of FKBP5 polymorphisms and childhood abuse on post-traumatic stress symptoms: Results from the National Health and Resilience in Veterans Study	Cohort 1 (*n* = 1,585) and Cohort 2 (*n* = 577)	Secure attachment style fully counteracted the significant interaction of *FKBP5* homozygous minor allele carriage and history of childhood abuse that was associated with greater severity of PTSD symptoms.	Results suggest that treatment designed to promote a secure attachment style may help counteract the deleterious interactive effect of *FKBP5* polymorphisms and childhood abuse and help mitigate risk for PTSD.
Pitts et al. (120)	Depression and cognitive dysfunction in older U.S. military veterans: Moderating effects of BDNF Val66Met polymorphism and physical exercise	Cohort 1 (*n* = 1,386)	The detrimental effect of depression on cognitive functioning was moderated by two factors known to alter BDNF function the brain: *BDNF* Val66Met genotype and physical exercise.	For veterans at risk of cognitive dysfunction, prevention and treatment efforts designed to promote physical exercise may help preserve cognitive functioning.

In addition to genetic and gene-by-environment studies, we examined how psychological factors may be linked to markers of biological aging, such as telomere length and DNA methylation age, in veterans. As with candidate gene findings, these findings should be interpreted with caution, as they may be tissue-specific (i.e., derived from cells present in saliva).

### FK506 Binding Protein 5

Examining the interactive effects of candidate genes and environmental factors on risk for mental disorders such as PTSD, rather than investigating independent genetic or environmental influences, may help advance understanding of the etiology of these conditions. Common single nucleotide polymorphisms (SNPs) in the FK506 Binding Protein 5 (*FKBP5*) gene may interact with childhood abuse to increase risk of developing PTSD (123–125). Results from Watkins et al. (113) suggested that the main effects of four *FKBP5* SNPs (rs9296158, rs3800373, rs1360780, rs9470080) were associated with lifetime severity of PTSD symptoms in veterans from C1 and C2 of the NHRVS. Results of this study further revealed that *FKBP5* polymorphisms, directly and interactively with childhood abuse, predicted greater severity of lifetime PTSD symptoms, specifically hyperarousal symptoms.

Building upon these findings, Tamman et al. (122) examined attachment style as a potentially modifiable environmental moderator of this association. A majority of individuals that experience abuse during childhood endorse insecure attachment styles (126), which has in turn been linked to increased risk for PTSD (127). Attachment style is also clinically relevant given that an insecure attachment style has been linked to reduced treatment response among veterans with PTSD (128). This study found that *FKBP5* SNPs, childhood abuse, and insecure attachment style were associated with greater PTSD symptoms (122). Importantly, *FKBP5* homozygous minor allele carriage and history of childhood abuse was associated with greater PTSD symptoms, but these effects were fully counteracted by secure attachment style (122).

### Neuropeptide Y

Another gene of relevance to traumatic stress and resilience is the neuropeptide Y (*NPY*) gene, which is expressed in a number of brain regions and plays a key role in the regulation of fear, stress, anxiety, learning, and memory (129). Previous studies have found that the rs16147 SNP, which is located in the promoter region of the *NPY* gene and accounts for more than half of the *in vivo* plasma expression of NPY, may interact with traumatic or stressful experiences to predict PTSD symptoms (130). Watkins et al. (117) examined whether polymorphisms in this gene may be linked to resilience to traumatic stress and PTSD symptoms in the C1 genetic subcohort of the NHRVS. Results of this study suggested that the T allele of *NPY* rs16147 was associated with greater resilience to PTSD symptoms, particularly re-experiencing/intrusive symptoms, even in veterans exposed to very high levels of trauma. Further research is need to evaluate whether interventions designed to enhance NPY expression levels, such as intranasal NPY (131, 132) may help promote stress resilience in trauma-exposed individuals (117).

### Apolipoprotein E

The apolipoprotein E (*APOE*) gene has also been implicated in PTSD risk. This gene is active in neuronal repair via cholesterol metabolism and transportation (133). This gene has also been associated with greater probability of developing neurologic and psychiatric disorders (133). Previous research examining the association between *APOE* gene polymorphism and PTSD risk has been mixed due to small and select samples. Accordingly, Mota et al. (118) used data from the genetic subcohorts of C1 and C2 of the NHRVS, and examined the relation between *APOE* genotype and PTSD symptoms. In both C1 and C2 of the NHRVS, the interaction of *APOE* ε4 carrier status and cumulative trauma burden was associated with higher PTSD symptoms, particularly re-experiencing/intrusion symptoms. Notably, they also observed an environmental moderation effect of social support, with greater social support associated with lower severity of PTSD symptoms among *APOE* ε4 allele carriers with greater cumulative trauma burden.

The ε4 allele of the *APOE* gene may also increase risk of cognitive dysfunction among normal aging veterans (134), especially given PTSD is associated with cognitive decline and difficulties (135). Accordingly, Averill et al. (119) examined the effects of *APOE* ε4 genotype and PTSD on cognitive functioning in veterans from C1 and C2 of the NHRVS, as well as a younger, predominantly civilian, replication sample from the Yale–Penn Study. Results revealed that *APOE* ε4 allele carrier status and PTSD were independently associated with lower cognitive functioning in the NHRVS samples (119). Specifically, veterans with PTSD who were ε4 carriers scored lower than those without PTSD, and the most pronounced differences were observed in executive function and attention/concentration. The significant interaction of ε4 and PTSD in predicting executive function was also replicated in the Yale–Penn cohort, but the main effects of ε4 and PTSD were not. Results of these studies suggest that *APOE* ε4 allele carrier status may contribute to the genetic etiology of PTSD symptoms and cognitive difficulties in U.S. veterans. They further highlight the role of trauma burden and social support in moderating the effect of ε4 on PTSD symptoms, and of PTSD in moderating the effect of ε4 on cognitive difficulties.

### Oxytocin Receptor

Polymorphisms in the oxytocin receptor gene (*OXTR*) may also interact with attachment style to predict PTSD, as the oxytocin system plays a key role in social behavior and stress regulation (136). Results from Sippel et al. (116) revealed that insecure attachment style and the interaction of the *OXTR* SNP rs53576, which has been implicated in empathy, loneliness, and parental sensitivity, and attachment style were associated with probable lifetime PTSD. Specifically, veterans with an insecure attachment style were at significantly increased risk of screening positive for PTSD if they had at least one rs53576 A allele, which has been linked to reduced empathy. However, the *OXTR* rs53576 genotype was not associated with PTSD when tested using a GWAS approach in a civilian sample. However, this GWAS did detect a new associated SNP (rs2300549), which was then tested in the veteran NHRVS sample, and while the main effect was null, there was preliminary evidence that it also interacted with attachment style to predict PTSD. Taken together, results of this study indicated that polymorphisms in the *OXTR* gene and attachment style may contribute to vulnerability to PTSD in veterans.

### Brain-Derived Neurotrophic Factor

Genetic studies from the NHRVS have also implicated a specific polymorphism in the brain-derived neurotrophic factor (*BDNF*) gene as a potential risk factor for PTSD (137, 138). BDNF is also know to influence synaptic plasticity, differentiation, and neuronal function. Prior research has found that Met allele of the Val66Met polymorphism of the *BDNF* gene is associated with impaired fear extinction, as well as reduced hippocampal volume and function, in individuals with PTSD (139, 140). Physical exercise has been linked to increased memory function, plasma BDNF levels, and hippocampal neurogenesis (120, 141). Physical exercise has also been linked to reduced depressive and PTSD symptoms (120, 141). To evaluate if physical exercise moderates the effect of the Val66Met SNP on risk of PTSD, Pitts et al. (141) examined the relationship between *BDNF* Val66Met Met allele carrier status, physical exercise, and PTSD symptoms in the NHRVS genetics subcohort. The authors found that relative to Val/Val homozygotes, Met allele carriers reported greater severity of lifetime PTSD symptoms and Met allele carriers with a higher number of traumas reported greater severity of PTSD symptoms (141). Greater engagement in physical exercise moderated this association where, among veterans with high trauma burden, Met allele carriers who engaged in regular physical exercise had significantly lower severity of PTSD symptoms relative to those who did not exercise (141). Another study examined the direct and interactive effect of the *BDNF* Val66Met polymorphism, depression, and physical exercise in predicting cognitive functioning in the NHRVS genetics subcohort (120). Pitts et al. (120) found that depression was associated with moderate decrements in cognitive functioning and this association was moderated by the *BDNF* Val66Met genotype and physical exercise. Jointly, results of these studies suggest that physical exercise interventions may help mitigate PTSD symptoms in trauma-exposed veterans and cognitive dysfunction in depressed veterans who are Met allele carriers.

### Genome-Wide Association Study of Depression and Alcohol Dependence

Alcohol-related problems, such as alcohol dependence, and MDD are other mental health disorders that are common in genetics research, as it is hypothesized that shared genetic factors may predispose individuals to both alcohol dependence and MDD. Using four genome-wide association study (GWAS) data sets, including C1 from the NHRVS, Andersen et al. (115) examined whether alcohol dependence and MDD have genetic overlap using polygenic risk scores. Polygenetic risk scores quantitatively measure the cumulative effects of common genetic variations across the genome in consideration of risk for a disorder. Results of this study revealed that higher MDD polygenic risk scores were associated with an increased risk for alcohol dependence (115). Results also suggested that there are common genetic factors which contribute to comorbid MDD-alcohol dependence, and that some individuals carry a genetic predisposition for both disorders (115). Findings from this study add a significant contribution to better understanding co-occurring disorders in veterans.

### Telomere Length and DNA Methylation Age

In addition to characterization of genetic risk factors for PTSD, alcohol dependence, and MDD, characterization of factors that may accelerate biological aging is important. Telomeres are nucleoprotein structures that cover the ends of chromosomes and protect from damage (142). Importantly, telomere length is associated with aging-related medical conditions and mortality, in addition to being an indicator of an individual's biological age (142). Hostility is characterized by aggressive urges/impulses and difficulties controlling anger (143). Research has established that hostility is prevalent among veterans (72) and is associated with aging-related disorders and telomere shortening (144). Using data from the NHRVS, Watkins et al. (114) found that greater severity of hostility, particularly difficulties controlling anger, was associated with peripheral telomere shortening in veterans.

Another study (112) evaluated whether negative age stereotypes, which have been linked to increased rates of physical decline, cognitive decline, and mortality in older adults (65, 145), are associated with shorter telomere length in veterans from the NHRVS and an independent sample of civilian older adults who recently experienced an acute myocardial infarction. In both samples, negative age stereotypes were associated with shorter telomere length, independent of sociodemographic characteristics and physical and mental-health indicators.

Aging has also been associated with predictable changes in DNA methylation. Genome-wide methylation research has established algorithms that estimate chronological age and serve as an “epigenetic clock” (146). These estimates, called DNA methylation (DNAm) age, can be used to quantify if DNAm aging is accelerated within an individual, which can predict detrimental health outcomes and is associated with sociodemographic, health, and psychosocial characteristics (121). Specifically, Tamman et al. (121) found that three physical health variables—diabetes, hypertension, and body mass index—were associated with accelerated DNAm aging. Cumulative trauma burden, child sexual trauma, and negative beliefs about aging were additionally associated with accelerated DNAm aging. Notably, child sexual abuse explained nearly the same amount of variance in accelerated DNAm age as diabetes (33.2 vs. 35.9%), thus underscoring the importance of trauma exposure in the acceleration of DNAm age. These results suggest that prevention and treatment efforts to mitigate deleterious effects of trauma exposure and negative beliefs about aging, which are modifiable, may help forestall accelerated DNA methylation aging in veterans.

### Clinical Implications

Collectively, results of NHRVS genetic and epigenetic studies underscore the utility of assessing, monitoring, and treating trauma-exposure, specifically childhood abuse and cumulative trauma burden, in veterans with certain genetic polymorphisms (113, 118–120, 122). They further suggest that therapeutic enhancement of modifiable protective factors, such as social support networks (e.g., one-to-one mentorship programs, peer support groups, social/relationship skills interventions, Vet-to-Vet programs), attachment style, and physical exercise among trauma-exposed veterans at elevated genetic risk for PTSD and related disorders may be an important aspect of prevention initiatives (116, 120, 122). Further, prevention and treatment efforts designed to reduce implicit negative age stereotypes, and anger and hostility may help mitigate acceleration of biological aging, and ultimately help reduce risk for age-related disorders among veterans (112, 114). As noted above, however, these findings must be interpreted with caution and require replication in larger, more diverse samples, as well as in tissues other than saliva.

## Conclusions and Future Directions

This narrative review summarized results of 82 original research studies that have been published to date using data from the NHRVS. These studies have covered six major topic areas, including post-traumatic stress disorder, suicidality, aging, resilience and post-traumatic growth, special topics relevant to veterans, and genetics. Collectively, the results of these studies underscore the need to develop and test prevention and intervention strategies that aim to enhance modifiable protective factors in veterans. Just as risk factors may have additive and interactive effects, such that having multiple genetic, developmental, neurobiological, and/or psychosocial risk factors may increase allostatic load or stress vulnerability, having and enhancing multiple protective factors may help promote stress resilience (147). Prior research prevention and intervention strategies designed to enhance these protective factors have been limited to community samples and have received little to no empirical support (4, 148, 149). Results of NHRVS studies published to date suggest that interventions that aim to assist veterans in building social connections and becoming better integrated in their communities, such as interventions that promote volunteerism (150) and reduce loneliness (151, 152) may help enhance resilience among veterans and warrant further investigation (153).

Extant research from the NHRVS has covered a broad range of health issues of relevance to U.S. military veterans. In future research in the NHRVS, including a new cohort of more than 4,000 veterans who completed a baseline NHRVS survey in 2019–2020, we plan to examine other conditions that may co-occur with PTSD, such as attention-deficit/hyperactivity disorder, traumatic brain injury, chronic pain, and dementia. Despite the inclusion of numerous potential correlates in the initial two NHRVS cohorts, there are other relevant factors, such as a broader range of potentially traumatic experiences that were not assessed in these cohorts, that may be differentially associated with PTSD and related disorders. Additionally, the assessment of substance use (e.g., cannabis and AUD), as well as suicidality and non-suicidal self-harm behaviors has been expanded in this new cohort.

Information on genetic, epigenetic, and modifiable protective factors, such as social connectedness, attachment style, physical exercise, have the potential to be combined to develop clinically useful risk-prediction models for PTSD and related disorders. Each of these factors alone may be weakly or moderately informative when considered individually, but a combination of these factors may provide more integrative approaches for assessment, monitoring, and treatment to mitigate risk for mental disorders that are prevalent in veterans, and promote better functioning and QOL in veterans. Accordingly, a goal for future NHRVS studies is to employ advanced data analytic approaches such as machine learning to identify key combinations of psychological, social, and biological (i.e., genetic) factors linked to key health outcomes in veterans.

Longitudinal epigenome-wide association studies (EWAS) are also being planned to examine the stability and predictive utility of epigenetic (e.g., DNA methylation) changes associated with PTSD and related disorders in the NHRVS genetics subcohorts. Although longitudinal EWAS are expensive and difficult to conduct, cross-sectional studies cannot detect the dynamic nature of epigenetic mechanisms impacting complex and evolving psychiatric disorders such as PTSD, making it difficult to ascertain whether the underlying causal effect is environmental or genetic (154). These studies may also provide insight into time- or condition-varying effects in veterans (155).

In summary, NHRVS studies published to date have yielded several important new findings regarding the psychosocial and genetic epidemiology of mental disorders, suicidality, aging, resilience, and post-traumatic growth. Specifically, veterans report exposure to a wide range of potentially traumatic events and a considerable proportion of veterans report experiencing PTSD, MDD, AUD, and related symptoms in their lifetime. However, a majority of veterans were found to be psychologically resilient in later life. Results suggest that initiatives designed to promote protective psychosocial characteristics (i.e., resilience, gratitude, purpose in life) and social connectedness (i.e., secure attachment, community integration, social engagement) may help promote resilience and growth in veterans, and may help mitigate risk for prevalent mental disorders in this population.

Given the nationally representative nature of the NHRVS, these findings can directly inform population-based prevention and treatment efforts in the broader U.S. veteran population. Many of the findings may also be applicable to the general U.S. adult population, as the majority of the NHRVS cohorts were comprised of non-combat-exposed veterans. Further research is needed to examine how demographic changes in the veteran population, including race and ethnic factors, older age, and a larger proportion of female veterans, may influence mental, physical, and cognitive health outcomes over time; and to translate findings from the NHRVS and other large-scale epidemiologic studies of veterans into novel and targeted prevention and treatment strategies to mitigating risk for major health conditions, and preserving functioning and overall quality of life in this population.

## Author Contributions

BF was responsible for the formal analysis and writing, and original draft preparation. JT, NM, IH-R, JK, and SS contributed to writing, review, and editing of the manuscript. RP supervised and contributed to the conceptualization, investigation, analysis, data curation, writing, review and editing, and funding acquisition. All authors discussed the results, contributed to the writing, and approved the final manuscript.

## Conflict of Interest

The authors declare that the research was conducted in the absence of any commercial or financial relationships that could be construed as a potential conflict of interest.
